# Genome-Wide Identification Analysis of the R2R3-MYB Transcription Factor Family in *Cymbidium sinense* for Insights into Drought Stress Responses

**DOI:** 10.3390/ijms24043235

**Published:** 2023-02-06

**Authors:** Mengjia Zhu, Qianqian Wang, Song Tu, Shijie Ke, Yuanyang Bi, Sagheer Ahmad, Diyang Zhang, Dingkun Liu, Siren Lan

**Affiliations:** 1 College of Forestry, Fujian Agriculture and Forestry University, Fuzhou 350002, China; 2 Key Laboratory of National Forestry and Grassland Administration for Orchid Conservation and Utilization, College of Landscape Architecture and Art, Fujian Agriculture and Forestry University, Fuzhou 350002, China

**Keywords:** *Cymbidium sinense*, R2R3-MYB transcription factor, stress responsiveness, genome

## Abstract

*Cymbidium sinense* represents a distinctive Orchidaceae plant that is more tolerant than other terrestrial orchids. Studies have shown that many members of the MYB transcription factor (TF) family, especially the R2R3-MYB subfamily, are responsive to drought stress. This study identified 103 *CsMYBs*; phylogenetic analysis classified these genes into 22 subgroups with *Arabidopsis thaliana*. Structural analysis showed that most *CsMYB* genes contained the same motifs, three exons and two introns, and showed a helix-turn-helix 3D structure in each R repeat. However, the members of subgroup 22 contained only one exon and no intron. Collinear analysis revealed that *C. sinense* had more orthologous R2R3-MYB genes with wheat than *A. thaliana* and rice. *Ka/Ks* ratios indicated that most *CsMYB* genes were under purifying negative selection pressure. *Cis*-acting elements analysis revealed that drought-related elements were mainly focused on subgroups 4, 8, 18, 20, 21, and 22, and *Mol015419* (S20) contained the most. The transcriptome analysis results showed that expression patterns of most *CsMYB* genes were upregulated in leaves in response to slight drought stress and downregulated in roots. Among them, members in S8 and S20 significantly responded to drought stress in *C. sinense*. In addition, S14 and S17 also participated in these responses, and nine genes were selected for the real-time reverse transcription quantitative PCR (RT-qPCR) experiment. The results were roughly consistent with the transcriptome. Our results, thus, provide an important contribution to understanding the role of *CsMYBs* in stress-related metabolic processes.

## 1. Introduction

As one of the largest TF families in plants, the MYB family plays a vital role in regulating phytochemical biosynthesis pathways and responses to abiotic or biotic stress [1,2]. The MYB protein is characterized by a highly conserved DNA-binding domain named the MYB domain, which is at the N-terminus. This domain usually consists of the range of one to four imperfect amino acid sequence repeats (R) containing approximately 52–53 amino acid residues, which acquire a helix-turn-helix (HTH) conformation and intercalates in the major groove of DNA [3,4,5]. According to the number of R repeats, the MYB gene family can be divided into four categories; MYB-related, R2R3-MYB, 3R-MYB, and atypical MYB (4R-MYB). Among these, the R2R3 type is the largest subfamily with diverse functions [6,7].

Research on the R2R3-MYB genes of model plant *A. thaliana* (*AtMYBs*) found 126 *AtMYBs*, divided into 25 subgroups. Of these subgroups, *AtMYB60* and *AtMYB96* in subgroup 1 (S1) were proven to play a dual role in drought stress responses through the ABA signaling cascade to regulate stomatal movement and lateral root growth, and *AtMYB60* was only regulated at the initial stage of drought stress but inhibited at severe drought stress [8,9]. *AtMYB33*, *AtMYB101* in subgroup 18 (S18), and *AtMYB44* in subgroup 22 (S22) are also involved in ABA-mediated responses to environmental signals, and *AtMYB2* in subgroup 20 (S20) controls the ABA induction of dehydration responsive genes [5,10]. Moreover, many studies in other species have also shown that the expression of R2R3-MYB genes could enhance the tolerance to drought stress. The relevant research on rice (*Oryza sativa*) showed that overexpression of *OsMYB2* could enhance the up-regulation of genes encoding proline synthase and transporters to adapt to drought stress. Similarly, *OsMYB4* causes high levels of several amino acids involved in stress adaptation. *OsMYB60* promotes cuticular wax biosynthesis on leaf surfaces to enhance plant resilience to drought stress [11,12,13]. R2R3-MYB TF in wheat (*Triticum aestivum*) *TaPIMP1* is involved in drought response by binding to the MYB-binding site and activates the expression of the genes with the Myb *cis*-element. Another wheat gene, *TaMYB70*, can also contribute to drought tolerance by targeting the gene with an MYB recognition site insertion in the promoter [14,15].

Orchids are renowned for their beautiful appearance and medicinal importance. *Cymbidium* is one of the earliest orchid groups to be cultivated, making excellent potted plants and cut flowers due to its extremely high ornamental and economic value. Commercially important hybrids have been cultivated for a long time in China and adjacent regions [16]. They are primarily distributed throughout the subtropics and tropical areas of Asia and northern Australia, which makes them adaptable to various habitats, including terrestrial, epiphytic, and saprophytic types [17]. The terrestrial types mainly rely on rainfall to obtain enough water for survival and growth. However, a dry climate certainly impacts their survival [18]. Some of the most important terrestrial orchids with high ornamental values include *C. goeringii*, *C. faberi*, *C. ensifolium*, *C. sinensis*, *C. kanran*, *C. tortisepalum,* and *C. tortisepalum*. Among these, *C. sinense* has a long history of cultivation in China, and it can withstand severe drought stress [19,20]. The recent whole genome sequencing of *C. sinense* [21] has broadened the spectrum to understand the stress-response mechanism of orchids. Many R2R3-MYB TFs play important roles in orchid plants, such as flavonoid regulation in *P. equestris* and *Cattleya hybrid* ‘*KOVA*’, floral aroma regulation in *Pleione limprichtii* and *C. faberi*, and environmental stress responses in *D. officinale*, *D. hybrid*, and *P. equestris* [22]. However, studies on R2R3-MYB genes of orchids in response to drought stress have rarely been reported.

In this study, R2R3-MYB subfamily members in *C. sinense* (*CsMYBs*) were identified by using bioinformatics methods. A total of 103 *CsMYBs* were found, and analyses of their gene structures and conserved motifs, phylogenetics, *K*a/*K*s ratios, collinear relationships, and *cis*-acting regulatory elements were conducted. In addition, we analyzed the expression patterns of *CsMYBs* under both well-watered and drought treatments and identified potential genes that can play important roles in drought stress tolerance. The results lay a foundation for investigating more roles of MYB transcription factors in *C. sinense* and other orchids.

## 2. Results

### 2.1. Identification and Phylogenetic Analysis of the R2R3-MYB Genes in C. sinense

**Figure 1:**
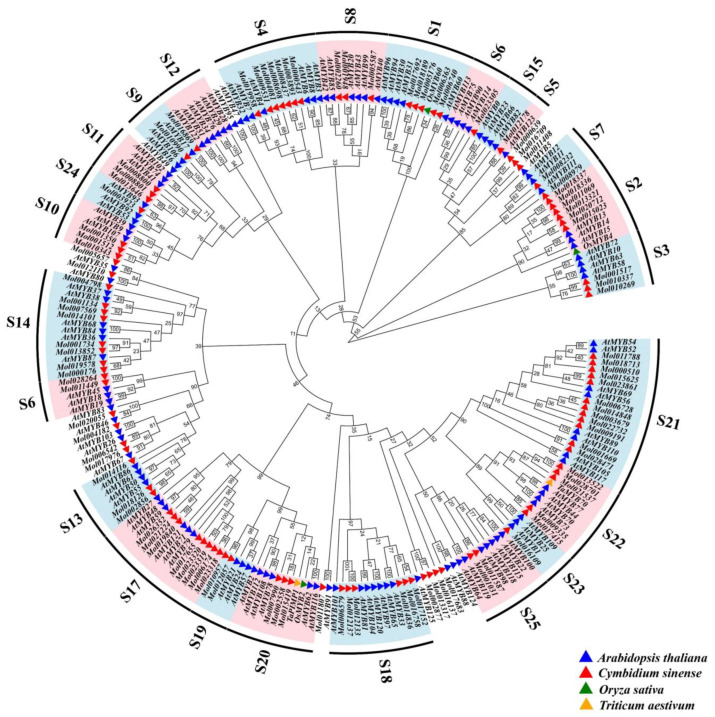
Phylogenetic analysis of R2R3-MYB proteins from *C. sinense*, *A. thaliana, O. sativa,* and *T. aestivum*. In total, 103 *CsMYB*, 126 *AtMYB*, three drought-responsive *OsMYB,* and two *TaMYB* protein sequenses were selected to construct the ML tree with 1000 bootstraps using RAxML-HPC2 on XSEDE. S1–S25 indicates the subgroups according to the classification of R2R3-MYB proteins in *A. thaliana*.

A total of 103 *CsMYB* genes (Table S1) were identified, as shown in the phylogenetic tree (Figure 1). The identified *CsMYBs* were divided into 22 subgroups according to Arabidopsis [5]. Of these groups, S21 contained the most R2R3-MYB genes, with 12 *CsMYBs*, followed by S14 and S17, each containing eight *CsMYBs*, while 11 drought-responsive R2R3-MYB genes (11 *DrMYBs*) from *Arabidopsis* (*AtMYB2*, *33*, *44*, *60*, *96*, *101*), rice (*OsMYB2*, *4*, *60*) and wheat (*TaPIMP*, *TaMYB70*) mentioned above were divided into S1, S2, S18, S20, and S22. Among these subgroups, S20 had only three members of *CsMYBs*, while others contained either five or six *CsMYBs*. In addition, no *CsMYBs* were classified into groups S12, S15, and S16.

### 2.2. Gene Structure and Domain Analysis of CsMYBs and DrMYBs

**Figure 2:**
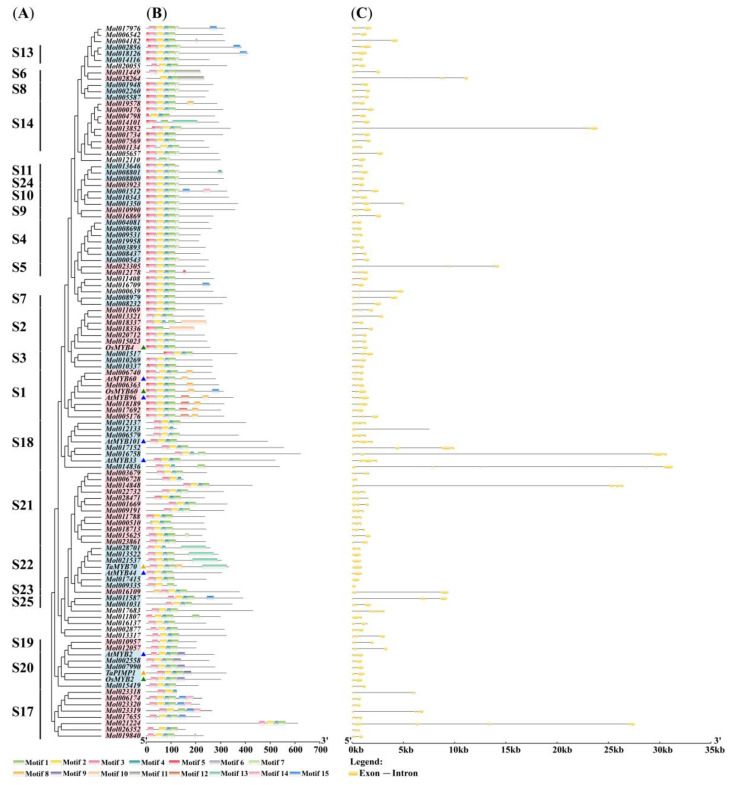
Phylogenetic relationships, conserved motifs, and intron–exon structure analysis of *CsMYBs* and *DrMYBs*. (**A**) Phylogenetic tree constructed with 103 *CsMYBs* and 11 *DrMYBs.* S1-S25 represent the subgroups. (**B**) Conserved protein motifs in different subgroups of *CsMYBs* and *DrMYBs*. The sequence logos of the motifs are listed in the supplementary files (Figure S1). The colored boxes indicate the motifs from 1 to 15, as listed at the bottom of the figure. (**C**) The predicted intron–exon structures in *CsMYB* genes and *DrMYBs*. The yellow boxes and black lines exhibit exons and introns, respectively.

**Figure 3:**
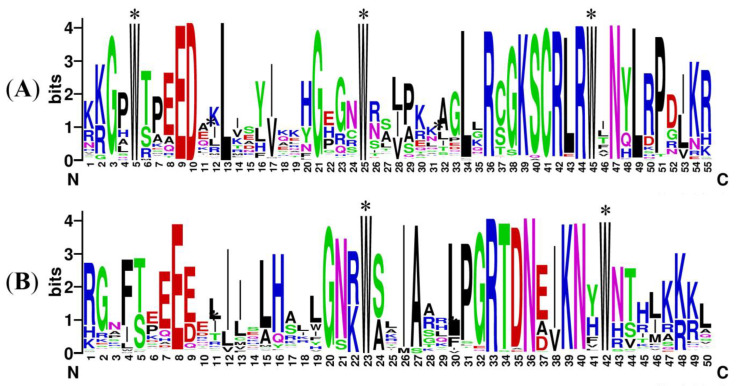
Sequence logos of the R2 (**A**) and R3 (**B**) repeats of *CsMYB* proteins. (**A**,**B**) indicate the logos of the R2 and R3 domains, respectively, based on multiple alignment analyses of 103 *CsMYB* proteins. * Indicates typical conserved tryptophan residues in the MYB domain.

To further understand the functional diversification of *CsMYBs*, the intron–exon structure and conserved motifs were compared with 11 *DrMYBs*, using GSDS software [23] and the MEME tool [24], respectively. As shown in Figure 2, 15 motifs ranged in length from 6 to 100. The results showed that most R2R3-MYB proteins in the same group contained similar motifs, further verifying their functional similarity. Almost all *CsMYB* proteins contained motifs 1, 2, 3, 4, and 6, which were part of the R2 (Figure 3A) or R3 domain (Figure 3B). However, motif 5 was present in more than half of the *CsMYB* members, which did not belong to the conserved domain. Additionally, motif 8 was present in all members of S1, *Mol019578* of S14, and *TaMYB70* of S22. Motif 13 was present in half members of S22 (*Mol028701*, *Mol013522*, *Mol021537*, and *TaMYB70*) and *Mol014101* of S14. Motifs 14 and 15 existed simultaneously in four members of S17 with close phylogenetic relationships. However, motifs 12 and 9 only appeared in S1 and S20, respectively. Moreover, *Mol018189*, clustered with *AtMYB96* in S1, contained motifs 8 and 12. These conserved motifs may be associated with the specific functions of the groups mentioned above. The exons in *CsMYB* genes ranged from one to 11. In S22, most members of *CsMYBs* only had one exon, similar to those of *DrMYBs* (*TaMYB70* and *AtMYB40*), while most of these genes consisted of three exons and two introns. These results showed the conservation of gene structure in *CsMYBs*.

To analyze the conservation of *CsMYBs*, aligned protein sequences were used to generate logos by WebLogo, as shown in Figure 3. A and B indicate the R2 and R3 repeats of *C. sinense*, respectively. Notably, some amino acids were more conserved, as tryptophan appeared at the sites of approximately every 18 amino acids. There were three tryptophans in R2 and two in R3.

### 2.3. Collinearity and Purifying Selection Analysis of CsMYB Genes

**Figure 4:**
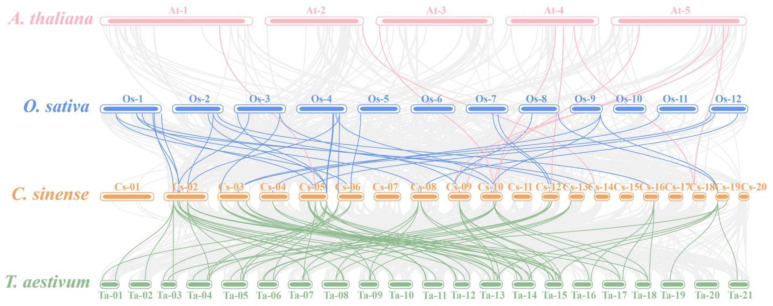
The collinearity of *CsMYB* genes between *A. thaliana*, *O. sativa*, and *T. aestivum*. The grey lines in the background indicate collinear blocks within the *A. thaliana*, *O. sativa*, *T. aestivum,* and *C. sinense* genomes, while the colored lines highlight the syntenic R2R3-MYB gene pairs.

*CsMYB* genes were unevenly distributed across 17 chromosomes in *C. sinensis* (Figure S2). To further analyze the potential evolutionary processes of the *CsMYBs*, the collinear relationships of R2R3-MYB genes were examined within *C. sinensis* and among *C. sinense*, *A. thaliana*, *O. sativa*, and *T. aestivum*. The results showed 15 linked regions within *C. sinensis*, and chromosome 10 had three R2R3-MYB gene pairs linked (Figure S2). For different species, 10 orthologues were detected between *C. sinense* and *A. thaliana*, 33 were detected between *C. sinense* and *O. sative*, and 73 orthologues were present between *C. sinensis* and *T. aestivum* (Figure 4). The Ka/Ks ratio reflected the selection pressure on the genes. In this study, we selected 36 pairs of *CsMYBs* with identity≥ 60%. Notably, four pairs, which were NaN (not a number), were excluded. The other 31 gene pairs scored less than 1, and most of the pairs (29/31) showed a value < 0.5; only one gene pair from S17 showed a rate greater than 1, which indicated that most *CsMYBs* mainly evolved under the influence of purifying selection. The divergence time of 32 *CsMYB* gene pairs was in the range of 1.31 mya for *Mol018337* and *Mol018336* of S2 to 178.98 mya for *Mol002856* and *Mol018126* of S13 (Table S2).

### 2.4. Protein Secondary and Tertiary Structures Prediction

The secondary structure prediction indicated that *DrMYB* and *CsMYB* proteins were both composed of α-helix, random coils, extended strands, and β-turns, with their means accounting for 35.94%, 8.15%, 5.65%, and 50.26% of the protein structure, respectively (Table S3).

**Figure 5:**
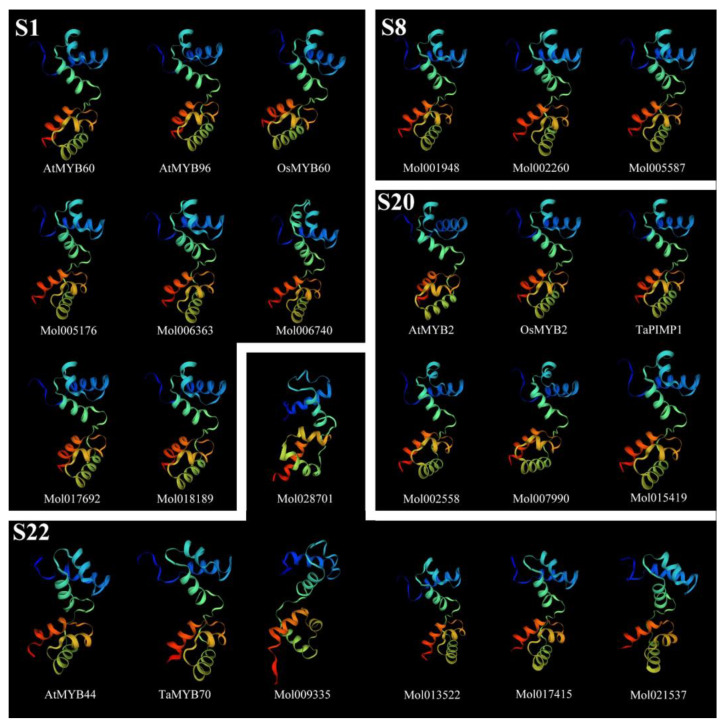
Protein tertiary structure of R2R3-MYB genes from subgroups 1, 8, 20, and 22. The tertiary structures were colored in rainbow order, representing N to C terminus.

Tertiary structures of a few members from subgroups 1, 8, 20, and 22 were highly conserved, characterized by six helixes, among which each R repeat forming three α–helixes, and an extended strand in the C terminus. The second and third helixes of each repeat build an HTH structure with three in R2 or two in R3 regularly spaced tryptophan residues. With the combination of secondary structure, members of S22 showed an extended strand in the N terminus, especially *Mol009335*, which were rare in other groups, and *Mol021537* (S22) exhibited very random coil helixes (Figure 5).

### 2.5. Cis-Acting Regulatory Elements Analysis in CsMYB and DrMYB Genes

**Figure 6:**
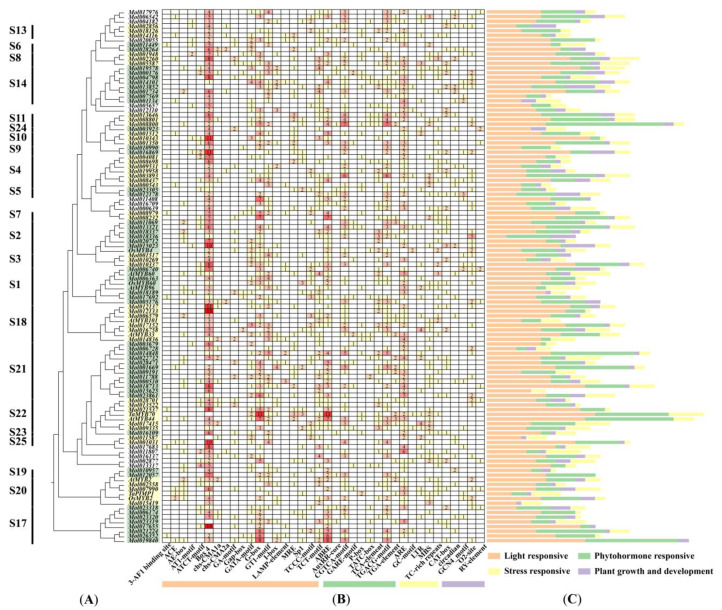
Analysis of *cis*-acting regulatory elements in *CsMYBs* and *DrMYBs*. (**A**) Phylogenetic tree constructed with 103 *CsMYBs* and 11 *DrMYBs.* S1-S25 represent the subgroups. (**B**) Heatmap of the number of *cis*-elements. Color bars and values in the box indicate the classification and number of *cis*-elements, respectively. Orange, green, yellow, and purple colors exhibit *cis*-acting elements in light responsiveness, phytohormone responsiveness, stress responsiveness, and plant growth development. (**C**) The sum of 4 types of *cis*-elements in each gene is represented by a histogram of different colors.

To better understand the functions of the R2R3-MYB genes in *C. sinense*, the 2000 bp upstream promoter regions of all *CsMYB* genes and 11 *DrMYBs* were predicted for putative cis-acting elements (Figure 6). A total of 38 kinds of elements were identified and divided into 4 types according to their functions, such as light responses, phytohormone responses, plant growth and development, and stress responses. Among them, light responsiveness comprised the highest proportion, 52.7% (1193/2265), followed by phytohormone-responsive functions (608/2265). The stress-responsive type (297/2265) contained 5 cis-elements and was composed of ARE elements (163/297), MBS elements (48/298), TC-rich repeats (40/298), LTR elements (39/298), and GC motifs (7/297) (Table S4a). Interestingly, MBS elements, which were predicted to be involved in drought inducibility, were found in seven *DrMYBs* belonging to S1 (*OsMYB60*, *AtMYB96*), S18 (*AtMYB101*), S20 (*AtMYB2*, *TaPIMP1,* and *OsMYB2*) and S22 (*TaMYB70* and *AtMYB44*). The MBS elements were absent from three *DrMYBs* belonging to S1 (*AtMYB60*), S2 (*OsMYB4*), and S18 (*AtMYB33*). *Mol015419*, a member of S20, had three MBS elements in the promoter regions. Moreover, half of the *CsMYBs* in S18 and S22 contained one or two MBS elements. Additionally, almost all members of *CsMYBs* in S4, S8, and S21 contained MBS elements, which might contribute to drought responsiveness (Table S4b).

### 2.6. Expression Patterns Analysis of CsMYB Genes under Drought Stress in Leaves and Roots

**Figure 7:**
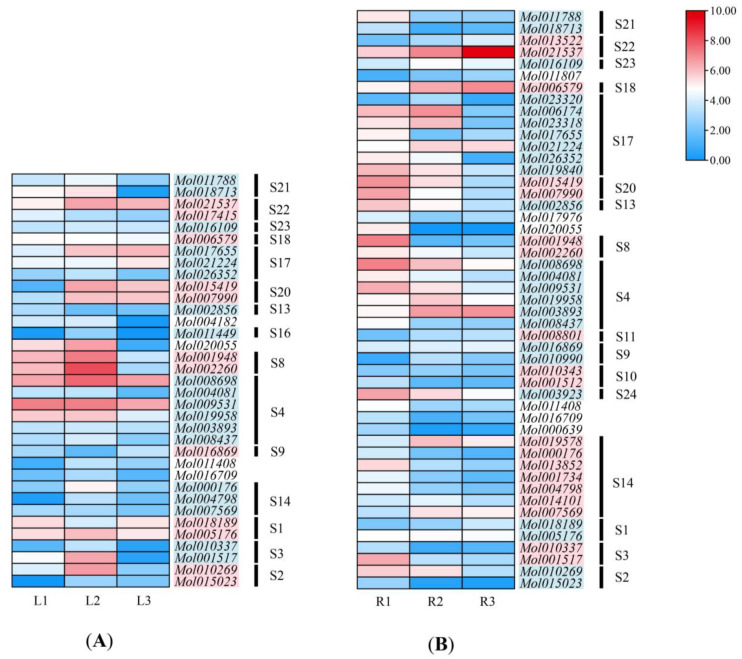
Expression profiles of *CsMYB* genes in leaves (**A**) and roots (**B**) of *C. sinense* under different degrees of drought treatment based on transcriptome data. L1, L2, and L3 represent control, slight, and severe drought treatments in leaves, respectively. The same treatments in roots are marked as R1, R2, and R3. The color bar represents the normalized FPKM values as follows: red, high expression level; white, low expression level; and blue, no expression. Detailed FPKM values are listed in Table S5.

To explore the expression patterns of the *CsMYB* genes, the transcript levels in leaves of *C. sinense* were analyzed (Figure 7A and Table S5a). The expression levels of *CsMYB* genes were evaluated by comparing the FPKM values for each gene under 3 leaf conditions (L1, L2, L3). Twenty-six out of 35 *CsMYBs* were modulated by drought stress, consisting of 16 upregulated genes and 3 downregulated genes in L2, and 6 upregulated and 14 downregulated genes in L3. Among them, *Mol015419* and *Mol007990* members in S20 were upregulated both in L2 and L3, with the most significant changes as compared to other R2R3-MYB genes of *C. sinense*. S3 (*Mol010269*, *Mol001517*), S4 (*Mol019958*, *Mol008698*), and S8 (*Mol002260*, *Mol001948*) were upregulated by slight drought stress and downregulated by more severe drought treatment (Figure 7A and Figure S3 and Table S6a).

In the roots of *C. sinense*, the FPKM values (FPKM > 5) in 49 out of 103 *CsMYB* genes were significant (Table S5b). Most of the detected *CsMYBs* (41/49) were modulated by drought stress. Only 10 out of 49 *CsMYBs* were upregulated, and 22 genes were downregulated by slight drought stress (R2). Under severe stress (R3), 31 genes were downregulated (Figure 7B and Table S6b). Notably, *Mol001948* from S8, which was downregulated 84-fold in R2 and 47-fold in R3, showed the most significant changes (Table S6b). Furthermore, *Mol021537*, a member of S22, was consistently upregulated during drought treatments, distinct from the patterns of S20, which showed consistent downregulation in roots. In addition, S4 (*Mol008698*, *Mol009531*, *Mol003893*), S8 (*Mol002260*, *Mol001948*), S14 (*Mol000176*), and S17 (*Mol023320*, *Mol017655*) also showed responses to drought stress in *C. sinense*. Interestingly, *Mol000176* (S14) showed a positive response to drought treatments through significant downregulation. Moreover, *Mol018189* (S1) was downregulated under slight drought stress both in leaves and roots, which was completely opposite to the expression patterns of *Mol010269* (S3) and *Mol001948* (S8) (Figure 7B and Figure S3).

## 3. Discussion

Many MYB TFs play important roles in orchid plants, but relatively few published studies have focused on stress-related responses. In this study, we identified 103 R2R3-MYB genes from the *C. sinense* genome, which is in line with the number of R2R3-MYB genes from other orchids, including 96, 99, 101, 102, and 104 R2R3-MYB genes in *P. equestris*, *P. aphrodite*, *D. officinale*, *C. ensifolium,* and *C. goeringii*, respectively. However, it showed a significant divergence compared to other species, such as *A. thaliana* and *O. sativa,* suggesting that several R2R3-MYB genes might have been lost in orchids during evolution (Table 1).

According to phylogenetic analysis, R2R3-MYB gene members of *C. sinense* can be divided into 22 groups based on *AtMYBs,* which are divided into 25 subgroups. Members with similar motifs or identical functions were divided into the same subgroup. *Mol018189* clustered with *AtMYB96* in S1, suggesting it might act through the ABA signaling cascade in response to drought stress. Moreover, *Mol021537*, a consistently upregulated gene during stress treatments, clustered with *TaMYB70* (S22), which confers enhanced drought tolerance of plants [15]. *Mol015419* and *Mol007990*, members from S20, clustered with drought-responsive *AtMYB2*, *OsMYB2,* and *TaPIMP1*, also showed positive responses to drought stress (Figure 1) [5,12,14]. The clustering results of *CsMYBs* and *AtMYBs* were similar to those of *D. officinale*, *P. aphrodite*, *C. ensifolium,* and *A. thaliana*, in which most members were found in the S21 subfamily [7,28]. There were no members of *CsMYBs* in S12, indicating that some special characteristics existed in the R2R3-MYB genes of *C. sinense*. In summary, the number and classification of R2R3-MYB gene members among orchid plants were similar.

Structural domain analysis of *CsMYBs* showed that the genes contained highly conserved tryptophan residues, among which R2 had three and R3 had two, while the first tryptophan residue in R3 was replaced by phenylalanine. This result is consistent with that observed in *A. thaliana* [6] and other plants. The substitution of the tryptophan residue in R3 may contribute to identifying new target genes and may result in the loss of DNA binding activity to target genes [30]. Additionally, the conserved motif analysis of *CsMYB* and 11 *DrMYB* protein sequences revealed close relationships in the phylogenetic tree and those with similar functions clustered into the same groups. Almost all the members contained motifs 1, 2, 3, 4, and 6. Interestingly, the members in S20 obtained motif 9, which was absent from other groups. The results might imply the special functions of the members in S20. The intron–exon structure analysis showed that the majority of *CsMYB* sequences (59%) consisted of two introns and three exons, while all members in S22 except *Mol028701* contained one exon and no intron except *AtMYB44* and *TaMYB70*. The secondary and tertiary structure predictions showed a highly conserved helix-turn-helix 3D structure in each R repeat of most R2R3-MYB proteins. Combined with the secondary structure, the members in S22 were characterized by a longer extended strand in the N terminus, which indicated that S22 might have some special functions in plants. In summary, the number of motifs, introns, exons, and protein structures in the same clade was similar or variable in a few groups.

Gene duplication is a main source of gene family expansion, playing a vital role in plant evolution. Three whole-genome duplication (WGD) events have occurred in *A. thaliana* [31], and *C. sinense* has experienced two WGD events [21]. There were only 10 collinear R2R3-MYB gene pairs between the dicot plants *Arabidopsis* and *C. sinense*, which was different from monocotyledons, such as rice and *T. aestivum* with more gene pairs, indicating that the R2R3-MYB gene family might have specific amplification between monocotyledons and dicotyledons. Furthermore, the number of collinear R2R3-MYB genes in *C. sinense* and *T. aestivum* was much higher than in rice. *C. sinense* experienced two WGD events of Orchidaceae plants together [32], and the *CsMYBs* may have expanded or rearranged in this event. The result of *Ka/Ks* analysis suggested that this gene family underwent purifying selection and highly conserved evolution. The results on *cis-acting* elements related to drought stress responses showed that the members of *CsMYBs* containing MBS elements were phylogenetically close to those *DrMYBs* with MBSs, which were thought to have an important role in the drought tolerance of plants. Moreover, the members containing MBS elements mainly focused on S4, S8, S18, S20, S21, and S22. *Mol015419*, a member of S20, contained 3 MBSs, displaying a positive response strategy to drought treatments according to the expression pattern. This indicated that the responses of *CsMYBs* to drought treatments might be highly related to *cis*-acting elements. Therefore, the study of *cis*-acting elements in the promoters of *CsMYBs* would provide significant value for further research.

It has been widely investigated in *Arabidopsis*, rice, and wheat that *R2R3-MYB* genes are involved in regulating responses to drought stress [5,11,12,13,33]. Among the other species, *GaMYB85* of *Gossypium aridum*, homolog with *AtMYB85* from S8, reportedly play an important role in the drought tolerance of plants [34]. R2R3-MYB gene *MdoMYB121* from *Malus domestica*, *MbMYB4* from *M. baccata,* and *PlMYB108* (S20) from *Paeonia lactiflflora* were involved in increasing drought tolerance [35,36,37]. Notably, these functional genes were detected in S8 and S20. Among the 103 R2R3-MYB genes of *C. sinense*, it was found that members of S8 (*Mol001948* and *Mol002260*) and S20 (*Mol015419* and *Mol007990*) showed significant expression changes under drought stress compared to other subgroups (Table S6). In addition, most *CsMYB* genes were upregulated in leaves but downregulated in roots. Notably, the slight drought treatment could initiate more responses to stress in leaves. However, in the roots of *C. sinense*, some genes exhibited different expression patterns in response to drought stress. *Mol021537,* a member of S22, was consistently upregulated during drought treatments, possibly due to the special gene structures with motif 13, one exon, and no intron. This result may imply that members of S22 possess some special stress-response mechanisms.

These findings screened out potential genes that can improve the efficiency of molecular breeding, contribute to the enhancement of the tolerance of orchids to stress in the future, and provide significant value for understanding the role of R2R3-MYB transcription factors in stress responsiveness. However, the mechanism of executive function in potential genes is still unclear. We next intend to verify these gene functions through transgene studies and protein interactions.

## 4. Materials and Methods

### 4.1. Identification and Phylogenetic Analysis of R2R3-MYB Genes in C. sinense

The genome data of *C. sinense*, previously described by Yang et al. (2021 [21]), was downloaded from an online repository (NCBI: PRJNA743748) to identify the candidate R2R3-MYB genes. For further verification, a blast was performed using R2R3-MYB proteins of *A. thaliana* (http://www.arabidopsis.org/, accessed on 31 March 2022) as a probe with an E-value of 1 × 10^-5^ by TBtools software [38]. The conserved domains (PF00249) were generated by a hidden Markov model (HMM) in the Pfam2 database. Those with two R domains (PF00249) were considered the R2R3-MYB subfamily, and the uncertain genes were uploaded to the NCBI website (https://blast.ncbi.nlm.nih.gov/, accessed on 31 March 2022) for a BLASTP search.

To explore the evolutionary relationship between the *R2R3-MYB* gene family of *C. sinense* and *Arabidopsis* and to better predict the functions of *CsMYBs*, 126 *AtMYBs*, five *DrMYBs* from rice (*OsMYB2*, *4*, *60*) and wheat (*TaPIMP1*, *TaMYB70*) and the identified *CsMYBs* sequences were aligned by MAFFT with auto strategy [39]. A phylogenetic dendrogram was constructed using the maximum likelihood (ML) method at the CIPRES Science Gateway web server (RAxML-HPC2 on XSEDE) [40]. The bootstrap values were 1000 replicates with the “PROTGAMMAAUTO” model, which predicted the fittest model and parameters. The generated tree was redrawn and annotated by EVOLVIEW [41].

### 4.2. Intron–Exon Structure and Conserved Domain Analysis of R2R3-MYB Genes

To further compare *CsMYBs* and 11 *DrMYBs*, the gene structures and the exon–intron structures of the protein sequences were analyzed by Gene Structure Display Server 2.0 (GSDS) [23], and the conserved motifs were analyzed by the MEME suite (Multiple Expectation Maximization for Motif Elicitation, version 5.1.0, University of Nevada, Reno and University of Washington, USA) [24]. The parameters were set as follows: 0 or 1 occurrence in each sequence; the number of motifs to be found, 15; the minimum width of the motif was 6; the maximum width of the motif was 100; and the motif must exist in all members of the same subgroup. Afterward, MAST XML was downloaded and redrawn with TBtools [38]. The R2 and R3 domains were produced by WebLogo (http://weblogo.berkeley.edu/logo.cgi, accessed on 3 April 2022) using aligned protein sequences of *CsMYBs* by MAFFT (Rozewicki et al., 2019 [39]).

### 4.3. Collinearity and Selective Pressure

The chromosome-level genomic Fasta files of *A. thaliana* were obtained from the TAIR database (http://www.arabidopsis.org/, accessed on 4 April 2022), and the genomes of *O. sativa* and *T. aestivum* were downloaded from the Phytozome website (https://phytozome-next.jgi.doe.gov/info/Osativa_v7_0, accessed on 4 April 2022) and NCBI database (https://www.ncbi.nlm.nih.gov/, accessed on 4 April 2022), respectively. The genome sequences of *C. sinense* [21] and the three species mentioned above were used for collinear block analysis of R2R3-MYB genes. In detail, MCscanX employed in TBtools was used to construct collinearity relationships between *C. sinense* and other species. The results were combined and visualized by the Dual_synteny_plot tool of TBtools [38].

To further analyze the selection pressure of *CsMYB* genes, two candidate gene sequences with similar genetic relationships were isolated according to the phylogenetic tree. DNAMAN software was used to compare gene pairs. The gene pairs that were more than 60% identical were used to calculate *Ka* (nonsynonymous substitution) and *Ks* (synonymous substitution). The *Ka*/*Ks*, *Ka,* and *Ks* values were calculated using TBtools [38]. Divergence time (T) was calculated by using the Formula T = *Ks*/(2 × 9.1 × 10^−9^) × 10^−6^ million years ago (mya) [42]. The Ka/Ks ratio was 1.0, critical for identifying genes under positive selection. Generally, *K*a/*K*s < 1.0 indicates purification or negative selection. *Ka*/*Ks* = 1.0 indicates a neutral choice, while *Ka*/*Ks* > 1.0 indicates a positive choice [43,44].

### 4.4. Protein Secondary and Tertiary Structures Prediction of DrMYBs and CsMYBs

The secondary structure was performed by the SOPMA program [45]. Tertiary structure prediction was performed and visualized by SWISS-MODEL [46] and colored by rainbow order representing N to C terminus.

### 4.5. Cis-Acting Element Analysis

The extraction of the promoter sequences was conducted using TBtools [38] to obtain 2000 bp upstream of *CsMYBs* and 11 *DrMYBs*. The online software PlantCARE (http://bioinformatics.psb.ugent.be/webtools/plantcare/html/, accessed on 7 April 2022) [47] was employed to identify and annotate *cis*-acting elements of promoter regions. The numbers and responsive functions of *cis*-acting elements were visualized by TBtools [38] and Origin software [48], respectively.

### 4.6. Expression Analysis of R2R3-MYB Genes under Drought Stress in Different Tissues of C. sinense

The experiment was carried out in a TR-YLD2000 climate incubator. The environmental conditions were set as follows: light intensity 25 μmol/(m^2^·s), light/dark (12 h/12 h), temperature (22–28 °C), and humidity (40%). First, healthy and mature *C. sinense* plants were selected to grow seedlings for 4 days with a well-watered treatment. On the fourth day, the control group was collected (L1 and R1), and the irrigation was stopped to initiate drought stress treatments. The light drought stress group was collected after three days without irrigation (L2 and R2), and the severe stress group was collected on the 7th day (L3 and R3) (Figure S4). Three replicates were collected for each group. The sampling was performed from 2–3 young leaves with a length of 3–4 cm, and newly grown roots were collected from the base of each repetition. The samples were collected in liquid nitrogen and stored at −80 °C for further use. During the drought treatment, the moisture matrix of each pot was monitored every day to detect the degree of stress.

Total RNA was extracted from different tissues using the cetyltrimethylammonium bromide (CTAB) method, and transcriptome sequencing was performed on the MGI2000 sequencing platform. The clean reads were obtained by removing low-quality reads and adapter- and poly-N-possessing reads (with >5% unknown bases) using SOAPnuke v1.4.0 software (BGI-Shenzhen, China) [49] and were mapped to the nucleotide sequences of *CsMYB* genes using HISAT2 version 2.1.0 (University of Texas Southwestern Medical Center, Dallas, TX, USA) [50]. The expression level of *CsMYBs* was calculated by the fragments per kilobase of exon per million fragments mapped (FPKM) method using RSEM v1.2.8 (University of Wisconsin-Madison, Madison, WI, USA) [51,52]. The heatmap of expression profiling was visualized by TBtools v 1.0971 (Chen et al., 2020). The genes with an FPKM value >5 in the control or drought stress treatment were regarded as sensible and were used to calculate the fold change (FPKM values of drought/FPKM values of control) [53]. Genes with a ≥2.0-fold change were regarded as upregulated genes, and those with a ≤0.5-fold change were defined as downregulated genes.

### 4.7. qRT–PCR Analysis

To verify the transcriptome data, several genes were selected for Quantitative RT–PCR (qRT–PCR) analysis. Three biological repeats were conducted. Specifically, total RNA was reverse transcribed into cDNA by TransScript^®^ All-in-One First-Strand cDNA Synthesis SuperMix for quantitative PCR (qPCR; TransGen Biotech, Beijing, China). The cDNA of each sample was diluted to 60 ng/mL, and 2 μL was used as a template for qRT–PCR. PCRs were performed using the Advanced™ Universal SYBR^®^ Green Supermix detection system (Bio-Rad, Hercules, CA, USA) in an ABI 7500 Real-time system (ABI, Foster City, CA, USA) with the following amplification regime: 95 °C for 30 s and 40 cycles of 95 °C for 5 s and 60 °C for 30 s. Actin from *C. sinense* (*Mol022529*) was used to normalize the expression of genes. The 2^−ΔΔCT^ method [54] was used to calculate the relative gene expression level. All the primers of *CsMYB* genes and actins for qRT–PCR were designed by Primer Premier 5 software and are listed in Table S7.

## 5. Conclusions

In this study, the 103 R2R3-MYB genes of *C. sinense* were identified from the genome. We analyzed their conserved motifs, exon–intron structures, secondary, and tertiary structures. The results showed that most R2R3-MYB genes were highly conserved, even from different species. Phylogeny and *K*a/*K*s ratios analysis indicated that most *CsMYBs* experienced negative selection, and three WDGs gave rise to more members in *C. sinense*. Functional *cis*-acting regulatory elements revealed that members in S4, S8, S18, S20, S21, and S22 might be drought-responsive, especially *Mol015419* (S20). We also analyzed the expression patterns of *CsMYB* genes in leaves and roots under drought treatment in wild *C. sinense*. Consequently, more genes were upregulated in leaves under slight drought stress (L2) but downregulated in the roots of *C. sinense*. Nine *CsMYB* genes, mainly from S1, S3, S4, S8, S20, S21, and S22, were used to verify the expression patterns at three water deficit stages in leaves and roots. The results consistently indicated that these *CsMYBs* were probably upregulated in leaves and downregulated in roots under slight drought stress. Among them, members of S8 (*Mol001948* and *Mol002260*) and S20 (*Mol015419* and *Mol007990*) showed positive responses to drought stress. Interestingly, members of S22, such as *Mol021537*, might have different stress mechanisms because of the consistent upregulation in response to drought treatments in the roots of *C. sinense*. The results will provide available information for further studies on the stress responsiveness of R2R3-MYB genes in different tissues of orchid species and other plants.

## Figures and Tables

**Table 1:** MYB transcription factors in nine plant species.—not determined; ^1^ this study.

Species		MYB Groups			
		MYB-related	R2R3-MYB	3R-MYB	Atypical MYB
Eudicot	*A. thaliana* [5,6]	64	126	5	2
	*P. trichocarpa* [25,26]	-	192	5	-
	*O. sativa* [26]	70	125	5	1
Monocot	*P. equestri* [27]	27	96	2	-
	*P. aphrodite* [7]	-	99	-	-
	*D. officinale* [7]	-	101	-	-
	*C. ensifolium* [28]	27	102	2	2
	*C. goeringii* [29]	-	104	-	-
	*C. sinense* ^1^	55	100	4	1

## Data Availability

The original contributions presented in the study are included in the article/Supplementary Material, and further inquiries can be directed to the corresponding authors.

## Supplementary Materials

The following supporting information can be downloaded at: https://www.mdpi.com/article/10.3390/ijms24043235/s1.
