# Supplementary material for: Genome-Wide Identification Analysis of the R2R3-MYB Transcription Factor Family in Cymbidium sinense for Insights into Drought Stress Responses
Source: Int J Mol Sci. 2023 Feb 6;24(4):3235. doi: 10.3390/ijms24043235 (PMC9959677; doi:10.3390/ijms24043235)
Supplement: Supplementary file 1 [file ijms-24-03235-s001.zip › ijms-2153948-supplementary.pdf]

Supplementary Material

Genome-Wide Identification Analysis of the R2R3-MYB Transcription Factor Family in *Cymbidium sinense* for Insights into Drought Stress Responses

Mengjia Zhu [1,2], Qianqian Wang [2], Song Tu [2], Shijie Ke [1,2], Yuanyang Bi [2], Sagheer Ahmad [2], Diyang Zhang [2], Dingkun Liu [1,2] and Siren Lan [1,2,*]

1 College of Forestry, Fujian Agriculture and Forestry University, Fuzhou 350002, China
2 Key Laboratory of National Forestry and Grassland Administration for Orchid Conservation and Utilization, College of Landscape Architecture and Art, Fujian Agriculture and Forestry University, Fuzhou 350002, China
* Correspondence: lkzx@fafu.edu.cn; Tel.: +86-139-0690-0395

SUPPLEMENTARY TABLE LEGENDS

TABLE S1| *CsMYBs* protein sequences used in the phylogenetic tree;
TABLE S2| Ka/Ks analysis of *CsMYB* genes;
TABLE S3| Protein secondary structure prediction of *DrMYBs* and *CsMYBs*;
TABLE S4a| Putative *cis*-acting elements identified in the promoter regions of *CsMYB* genes;
TABLE S4b| The number of MBS elements in *CsMYBs* and 11 *DrMYBs*;
TABLE S5a| Expression profiles of *CsMYB* genes in leaves;
TABLE S5b| Expression profiles of *CsMYB* genes in roots;
TABLE S6a| The fold-changes of *CsMYB* genes in leaves of *C. sinense* under drought stress treatment;
TABLE S6b| The fold-changes of *CsMYB* genes in roots of *C. sinense* under drought stress treatment;
TABLE S7| The primers of *CsMYB* genes;
FIGURE S1| The 15 motifs of *CsMYBs* and *DrMYBs*;
FIGURE S2| The collinearity of *CsMYB* genes in *C. sinensis*;
FIGURE S3| RT–qPCR validation of transcriptomic data of nine *CsMYB* genes under drought stress;
FIGURE S4| The plant of *C. sinense* under three treatments.

TABLE S1. *CsMYBs* protein sequences used in the phylogenetic tree

Gene ID	Sequence
Mol004798	MGRAPCCDKANVKKGPWSAEEDSKLKEFIEKLRWLNYLRPNIKHGEFSDAEDRTICTLFASIGSRWSIIASQLPGRTDNDIKNHWNTKLKK<br>KLLGISSSEKKNSLHHQQQQSQHLLFSSPTPSLFSNPYININATSAPIQIPFLSGFSHQANLVPISPEGFSASSTSSSSSSCLFQSSQYQVKERGST<br>VLAFGGESSDQGSCTQIRENMSLNNFLVYGNTGFQDFCGLQSSAPLEECLSYGFDEIKQLLMNSNNGIFLEDQTVEGCNSQWKSMSTS*
Mol014836	MSREINEGNRIEAPKDQFESSQNDESSCSRSSLAGGTVLKKGHWSPSEDAILVDYVKKHGEGNWNAIEKRTELRRCGKSCRLRWANHLKP<br>GLKKGALTREEVEKIDQLHCKLGSKWAKIAAMFCAWLILLEIILTASSSGIDSPKFMDALTYKPSHASDTSHFTVPNSILLPFPDSLHLLSENL<br>DSKGHSQAAAVRKQVTRESLARSHAVSNGLLPGRTDNEIKNYWHTRAKRYQRTCLPHYPQHVLRQALDENQQTLNYSMSNREDKGLGI<br>VLQSAMHDSAKFFKANPGPQAYSLPFSDISFTGLQSQDLVSHTYSFNPGVNHAIQLRETESYFPGNHGSMASGHAMFEQLPHGPNPVNKN<br>VPSFGYSEPGSLIQPNVNFSTSSPPTGTMKLELPSLQYPETDCYSRLAYPPPLPYEAIHTDIQSLPMVVSTQPGYTSQSSGLSETLVQVSHAIST<br>VGKQSSEKSSNYSAISPIDILENSSPNMCSADWKGSGDPNSPFSLPEVSPFSECLPIDDISFDDFVDSEATLGEHDLCLK*
Mol023318	MLPSIINQPRSMFMDNASIAATSFSISFIVGFAAAGQHGWGTFEDGWRKGPWTPQEDKLLTEHVKLHGEGRWNCVSRLTGLKRSGKSCRL<br>RWVNYLRPDLKRGKITPHEETIILELHAKWGNR*
Mol028264	MFILRQVDGVDLLIESIPHANPFRCRMRLRTCEQKVSVRKGLWSPEEDQKLKNYTAWPWMLELHSLSCRLRWINYLRPGLKRGIFTSEEED<br>IIMSLHAKLGNKWSRIAAHLPGRTDNEIKNYWNSYLKKKLMQQNHQHPMISSNELNESIQQTSKSENFQLESINQSATSSCSIDNGSFKSSFP<br>RVLFEEWLSPQDMGRDEFDENGLLEEMQSLDMFGEMIGDIDMNYDFIH*
Mol003923	MGRSPCCDENGLKKGPWTPEEDQILVLYIQKHGHGSWRALPKLAGLNRCGKSCRLRWTNYLRPDIKRGKFSPEEEQSILHLHSILGNKWS<br>AIATHLPGRTDNEIKNFWNTHLKKKLIQMGFDPMTHRPRTDFFAALPHLLALANLVDHRPSTWLADGLSAEASKLQCLQYILNAATPTSA<br>DNSMNNLLNSTDQSFPSSLDSMNSNQPLFNTHNTEIDVPLSYDQPLMSNENSGFSLFRHGEISTPVSALPPLTDLSNPGDACSTSSCGGSAAA<br>SFWPDLLLDDPFMAEFA*
Mol013646	MGRVPCCDKDGLKKGPWTPEEDQKLIDYIQKHGHGTWRTLPKNAGLARCGKSCRLRWANYLRPDIKRGRFSFEEEETIIQLHNILGNKWS<br>AIAARLPGRTDNEIKNYWNTHIRKRLLRNRPGDTPAPARSP*
Mol018713	MCSRGHWRPAEDEKLKDLVTKYGPHNWNAIAEKLPGRSGKSCRLRWFNQLDPRINRSPFTEEEEERLLTSHRIHGNRWSVIARLFPGRTD<br>NAVKNHWHVIMARKVRERSRFHGKRPCFSSSSLIMGDSSSNAQGKQGSLQEPQGFFSLVGNCYKKQQNCFLLGDFRSNSDEFQSWIHGFS<br>GDFSLVGKKNPIEFYDFLQVNSDSNGTKCCSSVEDEKDEADQQEQSKARPFFDFLPVNGSI*

Mol017692	MGRPPCCDKVGVKKGPWTPEEDINLVSYIQEHGPGNWKAIPTNSGLSRCSKSCRLRWTNYLRPGIKRGSFTEQEENLIIHLQALLGNRWAA<br>IASYLPERTDNDIKNHWNTHMKKKLRMENEGGMIAGSGFSKNQSIAKGQWEKRLQTDINMAKQALQEALSMEKPSFLYESKPSSSSHSSS<br>TPPSSTTYASSTENISRLLENWMRKAPKNSDKSTQHSVKNFVNIGADSTSSDGTITSVANNHIVSPEISQFQVDSKPCLDERFPLSLIETWLFD<br>ENYWGGNGSNNVLDFSFDDASHELF*
Mol011788	MCTRGHWRPEEDEKLKELVARLGPHNWNTIAEMLHGRSGKSCRLRWYNQLDPRINRCPFTEEEEERLLASHRIHGNRWSIIARLFPGRTD<br>NAVKNHWHVIMARRSRERSKLQTKQSSLSLESHLVRKEKKEIMNFEFSKGFLFSSLIGEGRSRTLCAFKHPISQSQEVHSYSPNFHEAEGDT<br>SMESSIEFYDFLQVKSDSNETGECSRREDERDQNEKEQQCEAGVPFIDFLAVGS*
Mol016109	MSSDADPMAVDETPPRPPPLENLASSSPPPPSSSPSASIECAGPQAEGFGMKSGAASRDRVKGPWSPEEDAILSRLVAKFGPRNWSLIARGV<br>PGRSGKSCRLRWCNQLDPQVKRKPFTEEEDRIIIAAHSLHGNKWAVIARLLEGRTDNAIKNHWNSTLRRKCIEIESCKRAPCEGQEDISADIL<br>EKTKGSSEETQSFGDVKPFTGTEVRDISSRESLSHHSEDRVNTVGPEFKQPPTISRPVPRLSAFSPYNPGFSHATSSLQSRSPLNAPHFRACLS<br>GPGDYKLFENMLWEPQVPSRCGHGCCGTKTRDQNTSSSLLGPEFVEFIEPPPILNREIASMASELSNIAWLKSGLQTGIYSSCQMNPSSSSCT<br>ASTC*
Mol019840	MVVTREGIRKGPWTEQEDLQLVCFVSLFGERRWDFIAKVSGLNRTGKSCRLRWVNYLHPGLKRGRITPQEERLILDLHSQWGNRWSRIAR<br>RLPGRTDNEVKNYWRTHMRKMAQESKRCPPLSSSTCSSESEQLEGDNGMEKNEQRSNMMPKNLEIKEEYEVKVYPMDQIWNDIAATLEP<br>TTTSGLSFESYGHEAGNVSCVSAAMASPVWENCAESLWKMDDDELCVGIKS*
Mol022732	MAFSFSSAPQFSTVETQGRRNEAVLILEEQRVKRSSDFEGLSEKNGRIEDQEGIELESGHSKLCARGHWRPAEDAKLKDLVAQYGPQNWN<br>LIAEKLEGRSGKSCRLRWFNQLDPRINKKAFTEEEEERLLSAHRLYGNKWALISRLFPGRTDNAVKNHWHVIMARRQREQSNAYRRRKPC<br>TSSKIFHKRMEAKCSNNVCSAESTVSSNNHETLCINRSFPVHGFLTRYSFPQQPQQFEYLFDTQGELVAGRSGCFERLFDSAIDMRQASPLIV<br>VPGIHHSGFSDSNSEASASDSAINNAFISEEAELESED*
Mol008801	MVRVPCCDKDGLKKGPWTPEEDQKLIDYIQKHGHGTWRTLPKNAGLARCGKSCRLRWANYLRPDIKRGRFSFEEEETIIQLHNILGNKWS<br>AIAARLPGRTDNEIKNYWNTHIRKRLLRSGIDPVTHQPRLDLLNLSSLLNTVLFNQSARLDASKLVDIKPHLNAKFLRIAVSLLQSQYQKQN<br>LLKHNLFQQSHPNCHQTITQPLPSSFLYNPVQFNNAGQWQYNEESSCNLSNLNYENMAQSMTGLLHGAFNAEIPSCQNLYSSLDSVLSTSV<br>SSVTLNNSSNIISSNIEDEKIPTAATSLISRFLSC*
Mol013522	MAANDVDRIKGPWSPEEDEMLQILVEKHGPRNWSLISKSIPGRSGKSCRLRWFNQLSPKVEHRPFTPDEDETIISAHRRFGNKWATIARLLS<br>GRTDNAIKNHWNSTLERKEAAAAAAVAWTSEERMMGALEDCRPIKRSNGARLCFSPGSPSGCDDTGPTKRNYPQPSSLQVGKVIKADAP

DSTTGKWNPCDPFTFLTLSLPGSSCGQNESSDNQKQTDPQLLEKKPPSVSMASPFSLEFLEALQEIIHQEVKNYMSGLEHRGILPPPLPPEEDS
KLNSVAKGIGISRIN*

Mol001350

MVRSPSREERGLKKGPWTPEEDQKLIDYIQKQGSHGSWRQLPRIAGLNRCGKSCRLRWTNYLRPDIKRGNFSEEEEKLIINLHSMLGNKWS
LISTKLPGRTDNEIKNYWNTHLKKKLLLMGIDPVTHRRRTDLELFANFPKLLSSSTDSCSLINPLMENSLGLQADTVQLARIHLMQVLIQVL
LKAASFSSSSSSNPNAYSNLVLNNLMSSSLPPLETFSNNSSSSSLPLETSAFGEPSQTPTNYQDFNVQNSENISKQLLFHSNTNDLFASSENHS
VDQKQDQIDLNETSAAKSVNTRYLDTLNLTDLDDTDLSWKDVLEPAETLHAKGSGSHCWLISPNTLSLLFCLVHSPCRSLLNRLLLSGGVL
T*

Mol010990

MGRSPCCEKVGLKKGPWTPEEDQKLLSYIEEHGHGSWRALPAKAGLQRCGKSCRLRWTNYLRPDIKRGKFSMQEEQTIIQLHALLGNRW
SAIATHLPKRTDNEIKNYWNTHLKKQLAKMGIDPVTHKPKSDALASADGHTRSTANLNHMAQWESARLEAEARLVRESKLRSSAPPSPFP
PQYLPQPPPPVSMPTAASPSVDVLGAWQGEWPKPVVNSQAGSHNIDLESPTSTLSFSENMLPSRIPGMGTANDSTNWKCLKKPGFSLDTAE
AFVNAEATSWLTGSCSGGFAAGFTGMLMGNTNKQNSTEGCDDSDIAGGSCVDVEEGEDEAEENKNYWNSIFNLVNSSSPSNSPPAVF*

Mol001512

MGRYPCCDEVGVKKGPWTPEEDQKLVEYIKENGHGSWRHLPKSAGLNRCGKSCRLRWTNYLRPDIKRGKFSEEEERLIIHLHSVLGNKW
SSIATRLPGRTDNEIKNYWNTHLKKKLLFMGIDPVTHRPITNIDLIANLPNLLSHTNLKNLAISCDQSLHQLHTDAAQFARIQIIQSLLHILSSP
HPTYPADILLQLMNTKLEGLSQLVAGQGLMPNNIEGLKQSSNGFDYQEIASKIMDDISKQGELFSSNTGGGAAAANYSFPSLVSSSPENISV
DQKQLEQIISSNSSSVAACSSSSTPFDAWDGLHFSDQDAAELGWKDILE*

Mol008437

MGRSPCCEKAHTNKGAWTKEEDERLITYIKAHGEGCWRSLPKAAGLLRCGKSCRLRWINYLRPDLKRGNFTVEEDELIIKLHSLLGNKWS
LIAGQLPGRTDNEIKNHWNTHIKRKLLNRGIDTQGTHHPIAKAATAAPSKPTSMSAANTQQSMAEDEDAHKNWQGLPDLNLELTISLPATS
QMPVASRDNSTQSTCLCYSLGFPSISLACSCNMLQS*

Mol014848

MLDDGRPSGVRIISDDGGDRRDAGYYGAAGKRRGVASKQPETRPRQGRLQRWSAKDRDLAAWRDRHGYRRGCWVNQQQSRSLHPPPP
LFPSERDGKTNETAFPLEQQQQPSLIGWGLEGFSGGSGSYGSEKNGQSEEHDGEHESGQSKLCARGHWRPAEDAKLRELVAQYGPQNWN
LIAENLEGRSGKSCRLRWFNQLDPRINKTAFTEEEEERLLSAHRLYGNKWALIARFFPGRTDNAVKNHWHVIIARKHRELSNSHRRRKASS
SISSSAQAFDKNLQVNCYNNACIVESTISRNIDESFSTCTDLSLNSYAYRSFPGDGFFTRHIHEKQPQQFQYLTGSDEKAVTGRYGYSQRPFD
SENDFRQVSPLVFVPGTYQSGYSDSNPEASATGSVVKKGNAFIVEEGDNDREKISLPFIDFLGVRAT*

Mol018189

MGRPPCCDKIGVKKGPWTPEEDIILVSYIQEHGPGNWRSVPTNTGLSRCSKSCRLRWTNYLRPGIKRGNFTDQEEKLIIHLQALLGNRWAAI
ASYLPERTDNDIKNYWNTHLKKKLIRKLEIDVETEGESSLKRGASISNYQSVTKGQWERRLQTDINMAKQALQEALSMEKPSFLYEIKPSCS

SHSSNNSRSPSSTYASSTENISRLLQNWMRKPPNSSSSSDRSNSAESTQISVTANSSTSVGKTVSFGNDKRSSIVSPETCSLFQVESKPSLEPEL
HFSQLETWLFDENYVGGTESSMNMLDLALDESELF*

Mol010343

MGRSPCCDEVGVKKGPWTPEEDEKLVEHIKKHGHGSWRHLPRNAGLNRCGKSCRLRWTNYLRPDIKRGKFSEEEEGLIIHLHSMLGNKW
SLISTKLPGRTDNEIKNHWNTHLKKKLLIMGIDPVTHRRRADLEFLANLPNLLSSSNSCNRMTNSWGSALQLQAADAANLAQVQILQGLLQ
VLIASSSNSNSSSNLNMTNLLGSSLAPLRNIADILQVSRQLEALQNGSFGLAHGSLPATTQMPNNYQTLPHQDQPEVNGNENQNLLFHSNAP
SLVSASPENTPSVSLKQEHINSNEISAANSESSTPFDPWDALSLSDPNGADLGWKEILE*

Mol001031

MSITPVEKFVMEKSERNPYAMKADTELRFYQRYYDDLLISSGDTSFINTSEISSSTVEAKRGKSSSSSWAVSSVLTSRTSVRGQWTAEEDNL
LVKLVKEHGVRKWSQISKKLVGRIGKQCRERWLNHLRPGIKETWTEEEEISLIETHKELGNRWAEISKKIPGRSENSIKNHWNATKRRLSSK
RKGGIRKTSKGKTSLLQEYIIKTTELLNNSTSPKQQILPTTQTKVSDLNFLNCEHYFNGSIEDQIDFIDTSLPQFNELFEKEGNGSNGEFCSDV
YLTHHMDEVPVAPEFVNLHGENLKDFLGDDMDLECKKDMDLMEMILTCSSQEYPYNYFGRSSSDSSLT*

Mol012137

MTPSVELKEEPAVTPPETSAAVAGTAEASQSLKKGPWTAAEDAILIEYVKKHGEGNWNAVQKNIGLQRCGKSCRLRWANHLRPNLKKGS
FSPEEEALILQLHAQLGNKWARMAAHLPGRTDNEIKNYWNTRIKRRQRAGLPLYPPEIQYQLALMNQKQGNLMLHQNQIRPKNFMSHISP
TTTTLFNHSGTSNNNTNYLLGNSNLNPPPHQLQSSTFSVKTELPSTQLFSCCAGDQKLLPPPVPLTIGEGNNVLFDDLYRESQGSGELFNEGL
FFEPLSINCITTAESMPPQLPPPSNSKHSIIDPPYKLENSDITIGMQVMKQPIETMVSQDVCNQPKMNMFHTTMMPAEKLLSDWCELSPSAGV
TEDDIRVEMQQLASTLSMNNDWNFELSSWQNMPDIR*

Mol003893

MGRSPCCDKAHTNKGAWTKEEDQRLVSYIQAHGEGCWRSLPKAAGLLRCGKSCRLRWINYLRPDLKRGNFTEEEDELIIKLHTFLGNKW
SLIAGRLPGRTDNEIKNYWNTHIKRKLLSRGIDTQTHHPITATSAAFLSSPEIYSAVPESAVTSDHKCHSGSSSGGGSGNCADLNLELSISLPY
YSSPQPSPYSDDASTAAAVRTPETCFCYRLGFRGGEACVCESLKEQVVSGLAMSD*

Mol017415

MDGGGGGEEKIKGSWSPEEDALLMKLVERHGARNWTLISAGIQGRSGKSCRLRWCNQLSPEVHHRPFTAAEDAIIVAAHGKYGNKWATI
ARLLPGRTDNAIKNHWNSTLRRRRRVEAATVSALSGPESESDSGGKRPCRRDDLILKVVEPMTPDAVDPATSLSLSPPGESSAAVAASAAT
APDAGTGGGWKEKGWLGNACVMSVMRKMIAEEVRRYMSGLRLDGGGGFYSIVKAESASNGQD*

Mol008800

MGRVPCCDKNGLKKGPWTPEEDQKLIDYIQKHGHGTWRTLPQNAGLLRCGKSCRLRWANYLRPDIKRGRFSFEEEEAIIQLHSILGNKWS
AIAARLPGRTDNEIKNYWNTNIRKRLLRSGIDPVTHRPRLDLLNLSSLLSTVLFTQPSRLDALKLLDNKPQLNTEFLRIAANLLMSQCQHEN
LFEHNQVQESQPTYHQTIPQQLSASLLYNPVEFSNTSQWQYNEASSCSLSDNLVTINNDLRYEASTVELPSDQNLYSDLDSVLSTPVSSMTM
NDSSTAFNNSIEEEKDSYHNDFFDFQISDMLDINEFI*

Mol004182	MGHHSCCNQQKVKRGLWSPEEDEKLIKYVTTHGYGCWSEVPDKAGLQRCGKSCRLRWINYLRPDIRRGRFSPEEEKLIISLHAVVGNRW<br>AHIASHLPGRTDNEIKNYWNSWIKKKIRKPNTGTCSSSSPQCSSVQILPTLIPGFNSLNQFEPLANQTLTNSKPNDSNPIFPSSPIPLFMFDSEC<br>GGTVAKEDHELLLDVGNLNLVDVWNTNDNQNDHQVLSPLSTFVDQNYLPPLVDSMVAMVPPPGCIGEECEASGSQECFEKNELSEWVEA<br>QQYPGFFIWDQTVQGQLGGDELPVSTPPTSNTDASVDSFPSTL*
Mol009531	MGRSPCCEKAHTNKGAWTKEEDERLVAYIKVHGEGCWRSLPKAAGLLRCGKSCRLRWINYLRPDLKRGNFTEEEDELIIKLHSLLGNKW<br>SLIAGRLQGRTDNEIKNYWNTHIRRKLLSRGIDPATHRPLQGPGDVSFVKADEKGASLIRKEDEQKSNNSSSSNSNSRSSSDEPKRWRCPDL<br>NLELGISPPSHQEEDSEEDLMREELGLCFRNGLLEFR*
Mol009191	MSSLQQRSEAPVLLLSSLWPQASFLTASESPSEREFVRSKESWSFNTSHDDLRLSDESMESRDLETKQSKLCSRGHWRPTEDAKLKDLVAQ<br>LGPQNWNLIAQNLKGRSGKSCRLRWFNQLDPRINRKAFTSEEEERLLSVHHHYGNKWSLIARFFPGRTDNAVKNHWHVIMARKQREHSIS<br>YRRRRLSSSTSFNPSPHLQTMEHNSSSGESTITNTREESISSATTRTVHGLVLSPHQKKAINFIMGAKSIGDQITSSDSTSKASAREAVVFSGSE<br>LDRERINPPFFDFLGVGAAEEKPLPVCVMPRERERES*
Mol004081	MGRSPCCEKAHTNKGAWTKEEDERLTAHIKAHGEGCWRSLPKAAGLLRCGKSCRLRWINYLRPDLKRGNFTEEEDELIIKLHSLLGNKWS<br>LIAARLPGRTDNEIKNYWNTHIRRKLMSSGIDPATHRPLNEQQEKTTISFIKGEEKVEEFEGREEGKKSSSNSSEEMSAWIRRRPEEENKQW<br>RCPDLNLELCMSPPSLKQQALCQEPVKSEELSLCFNCRLGVNKSSDCKCGSGFLGLRSGVLDYRRLETN*
Mol013321	MGLKKGPWTPEEDRILVSYIQRYGHGNWRALPKQAGLSRCGKSCRLRWTNYLRPDVKRGDFSKEEEETIIYLHAMLGNRWSAIAAKLPG<br>RTDNEIKNVWHTHLKKRLNPNQAMKDSKRGNRNSAMNKEMKKKDMEDQVSPACSRSEASSFVIATENCDVWWKEDSMDFLKELPELD<br>EILTSDAQSASQNNWAGEEEEEDSTGKNYDRTRSSNEEDIRFWMNLLAEAGNLECFK*
Mol019958	MGRSPCCEKAHTNKGAWTKEEDERLIAHIRAHGEGSWRSLPKAAGLLRCGKSCRLRWINYLRPDLKRGNFTDDEDELIIKLHSLLGNKWS<br>LIAGRLPGRTDNEIKNYWNTHIRRKLLNRGIDPATHRPISAASTSNLSISFSSSSSVTDKRNGVSRVSALLPRCPDLNLDLCISPPLEEQLQQDL<br>LEESNCGGEFLRLGSSFLLDYRSLEMK*
Mol005176	MGRPPCCEKEGVKKGPWTPEEDIILVSYIQEHGPGNWRAVPTNTGLMRCSKSCRLRWTNYLRPGIKRGNFTDQEEKLIIHLQALLGNRWA<br>AIASYLPERTDNDIKNYWNTHLKKKLKKLQLSGEDGGGRTVNPISNNHHQSFSKGQWERRLQTDIHMAKQALSEALSLDTMNLRSPSPPS<br>LGPANACTYASSTENISRLLENWMKSTPKSRSRTSLGSMSNVCSDSTTMESTTPTPAGQIMTSSAVQLGFRPAKIEPNGVLKEESKESSVVA<br>PPPLSFIENWLFDDLSVGNGGDDHQDLVDLPLSDASELFQD*
Mol017976	MGHHCCSRQKVKRGLWSPEEDEKLVRYITTHGHGCWSSVPKQAGLQRCGKSCRLRWINYLRPDLKRGSFTEQEERVIIDVHRILGNRWA<br>QIAKHLPGRTDNEVKNFWNSCIKKKLIAQGLDPKTHNLIPNTRPPYINSTSNIPHFPQNTCIPFTISSNIKETDSGRMMPLQTSSDALALHDSV

AISTFQYEDPDVMMSFKEHNSHEYNISASSSSLDHANILQISPNQPDFMDDHCCLWASSTPLEEAILADESKQNGERQGQQGEVQSLGDKV
DQFNEAMINGDASFDLELMESELMPCGSVFCSDNSMEQLQWEC*

METEKQNAIVEVDLRRGPWTVEEDLILTNYISKHGEGRWNTLAKCAGLKRTGKSCRLRWLNYLRPDVRRGNITVEEQLLILELHSRWGNR
Mol015419 WSKIAQHLPGRTDNEIKNFWRTRMQKHANQQCMPPLLERIRTVVDSSRAVMGVHTASASSSGRNGFSSSSATSNYDDHLDLKDMASTLE
EEGWPEFPAHGCEGSMLGFWEFSAMGPNMYA*

Mol026352 MAASEEMRKGPWTEQEDAQLVCYVHLFGERRWDFIAKVSGWRCGGGSRSLCFTGLNRTGKSCRLRWLNYLHPGLKHGRMTPQEEQLIV
ELHSRWGNRWSRIARRLPGRTDNEIKNYWRTHKRKKAQERQSSLLSPSSSSSSSSSSLNSIVMPDAKKA*

MVRAPCCEKMGLKKGPWTAEEDQILISYIHNHGHGNWRALPKLAGLLRCGKSCRLRWTNYLRPDIKRGNFTREEEDTIIALHQMLGNKW
Mol015023 SAIAAKLPGRTDNEIKNVWHTHLKKRLKKTDTETTQEPKRKTQIESNEETTTHSYSSIDVSCSGATESSTSSTVENSQNSMESLNHEFEEIDE
SFWTEILQMESNGDYNSIDSMATEEFSSDFMDETSLLSAGSRDEDDMNFWLRVFLQAEELPEI*

MEIEVHGGATPHSEEEMELRRGPWTLEEDLVLMNYISSHGEGRWNSLARCAGLKRTGKSCRLRWLNYLRPDVRRGNITPEEQLLILELHS
RWGNRWSKIAQHLPGRTDNEIKNYWRTRVQKHAKHLQCDVDSKQFKDVMRYLWMPRLIERIRAASGNSNAAQSVAGYGTLPGDDHTF
Mol007990 GAATGWGGHGEIVKPRPESPATTVSSSGAGGYEVTEKPKDGEGIQVNVPQHISGGLWPEASLPTPGYAHQGIPELEQYWGLGGDISDNLW
SMDDIWYLQQ*

MGRSPCCEKAHTNKGAWTKEEDERLIAYIRANGEGCWRSLPKAASLLRCGKSCRLRWINYLRPDLKRGNFTEEEDELIIKLHSLLGNKWS
Mol008698 LIAARLPGRTDNEIKNYWNTHIKRKLISRGTDPATHRPLHEAQANNTISFFKGENKIVEFVKREDERNKSSSSSSCCSSNSSEKLLIWRRSEED
DDEKMKWRCPDLNLELSISPPFEKKEDLCDYNVLNGEALIGLCFSCKLGVKNSSECKCGGVFLGLSRGVLDYRSLETN*

MGRGRAPCCQKVGLNKGSWTVEEDQRLISYINKNGHENWRALPKLAGLLRCGKSCRLRWINYLRPDIKRGNFTKEEEDIIINLHELLGNK
Mol010269 WSKIAAHLPGRTDNEIKNVWNTHLKKRLNLKNFTPPSPTSMTKLHDDSLSPTSSSTTSISLSHETNSYSIDEETPFLDEIDNIIDPHLDMWDIF
DDISISTSPPVMTITNDPNNTLDITMKECKQWLADLEEELGLYIDSVDDGDEIKGSEGEDLVRNYFHRGPSSPSSIRASRS*

MGRGRAPCCEKVGLNRGAWTPEEDMRLIAYIRKYGHGNWRALPKQAGLLRCGKSCRLRWINYLRPDIKRGNFSAEEEETIIKLHGLLGNK
Mol010337 WSKIASSLPGRTDNEIKNVWNTHLKKRLKSNEQSSSSPTPPIVSTTNPEDTQSSSSFSSSTTSISIEHKCSHPCQDHSTDIPLLPNLDALDALDV
DIEPKITDLADDLKDSIDEIIGIDIEPEVWNDVVFTEECHEAEEKRRWLAYLEEELGLFDDNVLASMMTRWWRWLWWVKGIP*

MGGSDTNEVLEEDNNRADAEEENVNDCTGQSNLCVRGHWRPEEDCKLKALVSIYGPQNWNLIAENLEGRSGKSCRLRWFNQLDPTINRS
Mol028471 AFTEEEEEKLMAAHRLYGNKWAMIARLFPGRTDNAVKNHWHVIMARKYREQSTAYRRRKLNQAMDRRSEEIIPNHPFSFSSSIADCSTYL
HLLPNDGKNSSLSHAGSYTREKLFDFLSGQNPSNGDKTKNFITRTWKGKGIIMKP*

Mol011449

MRLRTCEEQKVSVRKGLWSPEEDQKLKNYMLQYGHGCWSSIPAQAGLQRNGKSCRLRWINYLRPGLKRGIFTSEEEEIIMSLHAKLGNK
WSRIAAHLPGRTDNEIKNYWNSYLKKKLMQQNHQHPMISSNELNESIQQTSKSENFQLESINQSATSSCSIDNGSFKSSFPRVLFEEWLSPQ
DMGRDEFDENGLLEEMQSLDMFGEMIGDIDMNYDFIH*

Mol012178

MRRERACLPKEELRRGAWTEHEDKLLSDYITSHGLGRWRSLPEKAGLNRCGKSCRLRWLNYLRPGIKRGNITDEEEELIIRLHKLVGNRWS
LIAGRIPGRTDNEIKNYWNSYIKKKVAMDVSESHSYSKAATTTTAAAATTTASTTQLSFKSPCSEKANEASSVTSMVKSKTYEAEDLNTNS
AVLHEQHSSMKPEGHQQVCLPPSASSDFDSGFEFSFGELSNSSVFSCYGWRDAKSMACSSVIGEDCSSRRMEER*

Mol006542

MGHHSCCNKQKVRRGLWSPEEDDKLIKYISTYGHGCWSSVPRRAGLQRCGKSCRLRWINYLRPDLKRGSFSPYEETLIIELHRILGNRWAQ
IAKHLTGRTDNEVKNFWNSTIKKKLISQAVEGLSIPNLSNQFPLHPQDLVPISSFQFHISHFQDQSQPYNFPNLAQMYNPQTPQHNNMENPT
VPHFLQLSSSYGPVWPFYQQEQQQPHQTYNHEPAMLFDEINELQFFTKSKGSLLDKKVEIAIDNQLVVTDLECFSSLSSMPYLESSHGYNSL
MEYMHVLTGSDSLAEVTTEDHNSNDTFIQPYLLP*

Mol021224

MVLSRKKLKKKFRSLLAESVAADELKDSRKDALSAAEVNHELQIVRELLVSKSRRPKLLKRGNRGNKKPLLDDSKNGDGSCAARQGSVE
NEGSIDLKDKGKDKKRKRVEDGVLELEEGFKKEEQKLEKKKKDKQKKKKQREMKLKKKGKEEVEGKEETINVDEIENMNGVSKIPEVVP
VQVDESESKKVYVGGIPYYSSEDDIRSFFEDCGTVTEVDCMAFPETGKFRGIAILSFKKTQGKIPPPLSISSSDPADPSIASQAVSDVRPASLL
QPLTRSSVGLPQDPEFHHRCQFGLGSRLRTCPDPWTRAVHHCFMPSDLRAVIHGISERLRTQDDLFRPEGAAKRALALDGSDMGGFFLKTQ
PYKANRNQKSDFAPEIIDGYNRVYVGNLSWDISEDDLKQFFSDCKIASIRFGTDKETGDFKGYAHSKASSIVSSIHTKDKTRTLMVAMKEG
TRKGPWTEQEDLQLVCCVSLFGDRRWDFIARISGLKRTGKSCRLRWVNYLHPGLKQGRMTPEEEHLILELHSKWGNKWSQIAGKLPGRT
DNEIKNYWRTHMRKMAQEMKRNSQHSSSSSSSTSSSCLSLKTSTNGMENQQEVSFLLTMPEEKKRR*

Mol000543

MGRSPCCEKAHTNKGAWTKEEDQRLIAYIGAYGEGSWRSLPTSAGLLRCGKSCRLRWINYLRPDLKRGNFTEEEDELIIKLHGLFGNKWS
LIASRLPGRTDNEIKNYWNTRIKRKLLDHGIDPQTHRPINSATRPPVLVNPTADLFATTVITKLDERSHSEGSSSASCTIDLNLDLSIVSLPHSS
VDDTPLTHSVTTSTMTTKMAATPAICLCYSLGFRGSEECGCQAVQEQQAVIRYCSSIEMELRNYFS*

Mol001517

MDLGSNLLILGESDLVFLVLGDLQGELDLEGIGGGGDSGVRERESLSIKILTLCFNTWIEGKQERTQVMGRGRAPCCQKIGLHKGSWTPQE
DIRLISYIRKHGHGNWRALPKQAGLLRCGKSCRLRWINYLRPDIKRGNFTKEEEDTIIKLHGSLGNKWSKIASCLPGRTDNEIKNVWNTHLK
KRLRHNNHPTQSPTNMAARPEVDAPSSSSSGTAVTEGEELSFNKIEIPIEPQLDMWDILDSSLSPNLSPLPSPPPVEDDDILSDIPIDPDLWSDD
TNIDSKTKEVDNSVGGKSADESKNWLAYLEEELGLFDEVETKETVELISMQVGESMGSYFHKRPSSSSSTSLTTLVDLTVVMHSPWSN*

Mol006174	MEGQYGWGTFEDGWRKGPWTPQEDKLLTEHVKLHGEGRWNCVSRLTGLKRSGKSCRLRWVNYLRPDLKRGKITPHEETIILELHAKWG<br>NRWSTIARSLPGRTDNEIKNYWRTHFKKEKTMKNIERARANFLKQQQEQKQQQQHQQLQLEVPQFEEIAITQLMQEIAYMCNMPSVFQDE<br>EFTSKSMVSSDDRLGDGEAYYATSENLWNLDDLGLDMGDQALPFYY*
Mol020712	MVRAPCCERVGLNKGQWTPQEDKILITYIQTYGHGNWRALPEKAGLLRCGKSCRLRWTNYLNPGIKRGNFTKEEEDTIINLQQILGNRWS<br>AIAAMLPGRTDNEIKNVWHTHLKKRLNPNQILQNSKRKIERRDNRSNSVIEFSSTSASESCSDFSSPTTESSSNVSERDNSNSRNDESTCDNG<br>VEEIEESFWTEILFVNGCDSPLMEVDSSKNEDLSFWLRIFMEAETLQGFPEI*
Mol002558	MDMRRGPWTMEEDLILMNYIAMHGEGRWNSLARCAGLKRTGKSCRLRWLNYLRPDIRRGNITPEEQFLILELQSRWGNRWSKIAQQLPG<br>RTDNEIKNYWRTRVQKHAKQLQCDVNSKQFQDVMRYIWMPRLMERISVASGELPAAECMNLTQTDQTIGCKQCETGPEISGCTGSCSEST<br>GSIGTTSTEFGKPECDRLSGSSGTHNHDVCWPEAAPQNPTGYAYEQELPELDQYWGGEEYENMWSMEDIWQLTQ*
Mol012057	MVDESSNADKRIHINQEAEVRKGPWTMEEDLILINYIANHGEGVWNNLARSAGLKRTGKSCRLRWLNYLRPDVRRGNITPEEQLLIMELH<br>ARWGNRWSKIARQLPGRTDNEIKNYWRTRIQKKVKNGDAPDYSSQMIIDEASTSHTSSIEEGMAQQSYPIHPNTNPEAYPPSCNSGELGEN<br>FWSMEDFWPIQSDQSLNGD*
Mol014116	MVSHSCCLKQKLRKGLWSPEEDEKLFNHIIRFGVGCWSSVPKRAGLQRCGKSCRLRWINYLRPDLKRGSFSQEEEDLIISLHEILGNRWSQI<br>ATHLPGRTDNEIKNFWNSCLKKKLRQNGIDPSTHKPLNEEQNEDKSHNLNDSNQMNSAMRPVFDPFPALDFQPGTVLNTYDQLQQPPLIP<br>ASDSLNASENYFYGESSASCINWNCSIGAELNFSSDSSYYYSSFQMKSSVFQNPSDACLDIPQSEFGGDLL*
Mol006363	MGRPPCCDKAGIKKGPWTPEEDIILVSYIQEHGPGNWRLVPTNSGLMRCSKSCRLRWTNYLRPGIKRGNFSSHEEEIIIQLQALLGNRWAAI<br>ASYLPQRTDNDIKNYWNTHLKKKIKKYQASVGSYITSSDSAGSTSHERVLGGYGADEIRNSCSSNSHTLTHSSSTYASSAENISRLLEGWM<br>RSSPNQTLNDKKLQHQMKKESITFNSNSFIMNQLNFDVEVNSQLPLEGSVIAWDKTATDSVFEENKQFKMMTYDDNKLKTQSQQPAMSV<br>LEKWLLEENPGQMDAFLETMF*
Mol017152	MERQRKRMTLGDEVMVLLRVAHNMTCSENDGDTLMLLKEQLESSSTDEGSCCGSSTTGGGRVTLKKGPWTTAEDALLISYVRKHGEGN<br>WNAVQRHSGLLRCGKSCRLRWANHLRPHLKKGAFSPEEEQFIIQMHSKMGNRWARMAALLPGRTDNEIKNYWNTRVKRRSRAGLPLYP<br>PKLSLQTSNENQLIQSASELSSGNHQNIGVLQGTMNGTLEFRCHPHKANSGPLPSPPRFPDIPVTDLPCHGLGYYTRSFLNSGANHFKQTWE<br>SETFSPGCHVNQNSIMVPAFEELPSNSETVYLNTGLGYPYDPDPENKNLASFICSTPGNPGNFSSSRLLPGAVKMELPSLQYPEADYIDWTSS<br>SLLKSSYDAVDMYIQSPENVSVHSDCASPRNKGLLEDLVQESHAISSGKKQSFEKSFDRAVAAVMASGTVKWEEIRDPTSPFCLSAVSLFN<br>DCNPTIISTFDESSACETPMGDKQDKYTPNLPYFLRPVLLGSGWLMGNSGDAEDSIAGALDENLHKERAPVPIPVPAGISSMASLEAYPWTN<br>MPRVCQMSEH*

MGRSPCCSKVGLHRGPWTAREDALLTSYVQNHGEGNWRSLPKLAGLLRCGKSCRLRWMNYLRPDIKRGNIGPEEEDLIIRLHRLLGNRW
Mol016709 SLIAGRLPGRTDNEIKNYWNSHLSKKLKKQGLKLREATPRTKQSNSSSNNKKQSTKIINQSETLGGTNETKIYAPKPTRLTSRIHVMENSSEE
EKGSSDEMELSANYLSDGGEHDFLNTNQYLDLGFDQFLPLQQDSVMFERLYDEYSQLLQSEVNDVNLFAYDQYLMV*

MERNSCCFKEGINKGAWSSTDDKLLTAFVNAHGEGKWTTVPSKAGLKRSGKSCRLRWLNCLRPNVKRGNFSEEEDDLIIRLHKLLGNRW
Mol023305 SLIAGRLPGRTDNEIKNYWSTTLSKKASFRHLIRQSSATKRPPASNREIPTKQSAEESTAKMVDEMPENSKGGCIVKEQQFQVEENMALNFG
SFDDDTMVALRDQTLMEFNGPMDFENWMLNDEYVDYLPDADQIQSLPSLFDIGGEF*

MVRAPCCEAMGLKKGPWTHEEDNILVSYILTNGHGNWRALPKKAGLLRCGKSCRLRWTNYLRPDVKRGDFSKEEEETIIYMHAMLGNR
Mol011069 WSAIAAKLPGRTDNEIKNFWHTHLKKRLNPSQFIKKTKPRTANPDEPKEEDKFEDQISPVFIQNESYFSENITESNSVDMKQNSIDSFEEYFH
DFDEILWSEEFTVTGEEEDGSIGLNFGKPSLSNEDSISFWLNLLAESGNLE*

MMGDQEEPNSVDKMINNSNEEAEVVRKGPWTMEEDLILMNYIAIHGEGFWSNLARSAGLKRTGKSCRLRWLNYLRPDVRRGNITPEEQL
Mol010957 LIMDLHSRWGNRWSKIARQLPGRTDNEIKNYWRTRIQKKVKDGETVNCSSQMMKYEGSSSHTGGLEEIIAQTSYPIYPNANPQEAYVPTYS
ESNPNETYLSAEDFWSIQSFNGL*

MEVQYGWGTFEDGWRKGPWTPEEDKLLTEHVKLHGEGRWNCVSRLTGLKRSGKSCRLRWVNYLRPDLKRGKITPHEETIILELHAKWG
Mol023320 NRWSTIARSLLGRTDNEIKNYWRTHFKKESTTKNIKRARAKFLKQQQHQQLQLEVPQFEEIAITQLMEIAYMCNMPYVFQGEEFTRKSMV
SSDNRLGDGEAYYATWENLWNLDDLGLDMGDQAVFYY*

MGRAPCCDKANVKRGPWSPEEDEALRSYIQNHGSGGNWIALPKKAGLKRCGKSCRLRWLNYLRPDIKHGCFTKEEDDIIFTLYSKIGSRW
Mol007569 SVIASKLPGRTDNDVKNHWNTKLKKRMMETQARLSGSHYRSSAPPLMASEPLALTDHSLASINCASIRPTDQIESQSFNGSFDEPLDLRVPR
QNPSFLDLDFGSITDLLCSSTYEETIETIWADASEEIKLGELSQSVAYLY*

MGRAPCCAKQGLKKGPWTPEEDKILVDYIQSNGHGSWCSLPKLAGLLRCGKSCRLRWTNYLRPDIKRGPFTSEEQKTIIQLHGIVGNKWST
Mol016869 IASQLPGRTDNEIKNYWNTHLKKRLLRMGINPDTYAPASPSSSAGGGGAGFPVTRHMAQWESARLEAEARLSRESLLFSSASSAAAVSAA
DSEPADDSLPAARKPESDFFLRIWNSEIGDAFRKPLPSTLPRSAAEEATTAPTEESKSCASAGGEAPSAPDSSSSNELDETSDRRTSST*

MGRHSCCHKQKLRKGLWSPEEDEKLLKHITKYGHGCWSSVPKLAGLQRCGKSCRLRWINYLRPDLKRGTFSQQEENLIIELHAVLGNRW
SQIAAQLPGRTDNEIKNLWNSCIKKKLRQRGIDPNTHKSLSETENPDENKVSTSSERNSILYTGDSLKQTVPQLTSSLMQSVDKNSIPVKEFC
Mol018126 IDNATTCLRSSSNSISSFPLPQLNYGTSATASLPLSSNPFLWFNQNCRVFDMNPELSCTSMSTMGPSISSSVLTNSIDLKSPVNLSSSCLPPTCT
GAPIFSYLDAGNPSRCSSGSSSSSSFFDSGFLSWPDLTTEKEIQINFQGDPEELKWSEYLQGNFPVSSVLHSHNQPLYGGDANSESHFDIEGV
GLWHQNQQPQHQQHQQQPQQQASDLYGKEYQRISTLFGQI*

Mol000176	MGRAPCCDKANVKKGPWSPEEDAKLKSYIEKHGTGGNWIALPQKIGMKRCGKSCRLRWLNYLRPNLKHGGFSEEEDNIICSLYVTVGSR<br>WSLIASHLPGRTDNDIKNHWNTRLKKKLLGKSRKDHQHHSRRIPKQLEPQKSEETNDFYWLDPALPPYPETDSQASIKKVLMKLGGRISID<br>EEAEIPPLINAPDHNSIYCSSNTSLMASSAQLSSFNECLSTNLNDIFASNEVKLEEFDNYSLYGMTTMAEEITGLLGMPEAVNWSTSDVSSSI<br>YSPMASTSVSYHGSMQQYVYQDHQMHLGMELSFKYE*
Mol002856	MKFYKRMGRHSCCYKQKLRKGLWSPEEDEKLLEHITKYGHGCWSSVPKLAGLQRCGKSCRLRWINYLRPDLKRGTFSQEEENLIIELHGV<br>LGNRWSQIAAQLPGRTDNEIKNLWNSCIKKKLRQRGIDPNTHKPLAEIDEQREKIAEIDSSGHPTPEPAKQTEQPAETAVLNNFVRPANSSA<br>ASYYSLPNLTYSDECGNNIGLIQQFWFNQSSKFFNTINPNSEFSFNSVSSLLPSVPRSTLSTSKELKPLTNLLMENSPSGFYWEAGNSSNSSAS<br>SGSNDPLFDGSIFPWTQLMPERDTNIQLHGETEDLKWSEYLNGSIPLSTDLQSQNPELCNGVKVEEQFGIDGLSIWLQNHQQQQQQQQQQ<br>QASNFYGKDCQKDMVC*
Mol008979	MGRAPCCEKVGLKKGRWTAEEDQLLVKYIKTNGEGSWRSLPKKAGLLRCGKSCRLRWINYLREDLKRGNISKEEEDTIIQLRANLGNRW<br>SVIAGHLPGRTDNEIKNYWNSHLSKKVEGLHDKEDENCDATGVTKVRKRKGGRPSKSAKKKKAEAVISSMDEGTKLKDVMEASKSEQGE<br>SIVTDLEPQELSLEDLPSIEILLVDEENIEGGRALNPNNEEEGKQKEDSGVIVEVLSNEEGGSSFDEIEKLVDWELEELALRLWEEGEGVGEV<br>TPWVWEDESPRINGEFGDFFDYEKFDAFKEDDYLDDWLLSDASECSSWFSSCG*
Mol008232	MVRAPCCDKKGLKKGRWTVEEDETLMNYIAENGEGSWRSLPKNAGLLRCGKSCRLRWINYLRSDLKRGSISKEEDEIIIKLHATIGNRWS<br>VIAAQLPGRTDNEIKNYWNSHLSRRVHCFRRHGESKTFIYDIGSIPSPGKRRGGRTSRAAMMKNSSSSMVSGSIREDQISPNPNQNQVLNHT<br>NNNLCISSSSSALTEKENSSLTFDFDFDLEEVLGPNGDIDAVSLEANEMGESRRATCSGEKEVELAMEEESGTMMTEMEKMLKWDFESLEA<br>KLWEEDGSDMWPWLWDISGDIIFDEEPLGSWLLA*
Mol019578	MGRAPCCDKENVKKGPWSPEEDVKLKSYIEQYGTGGNWIALPQKVGLKRCGKSCRLRWLNYLRPNLKHGAFSEEEDNIICSLYIRIGSRW<br>SLIAAQLPGRTDNDIKNYWNTKLKKKLLGKSSHKNHHQSQYTRRAPKQDPNNLQEIINTVEISNGSTSSASYWIQPATPTYMSSSTEIKRFLP<br>KLEGVPPLITPDHSDLNDTNSYLLEASPQPSSFTNCLSSSSTELDDIFGHFDSFYGSNGMSGEITGLLSMPDQVFNSLMYPPMAVSAKASYQG<br>TIGANGGLV*
Mol000639	MGRAPCCSKVGLHRGPWTSREDALLTSYIKNHGAGSWKSLPKRAGLLRCGKSCRLRWMNYIRPDIKRGNISPEEEDLIIRLHSLLGNRWSL<br>IAGRLPGRTDNEIKNYWNGHLSKKLRNQGFAMRAAVSRRPKPSRDHSSMKKTNNTTTEMKSTSEKATVSKIYAPKPTRIERRYRNYVMSA<br>VTESNEETEGSDGRLDSGEESSSENYQENISCSDNGEEANLLDVNRYLGLDLDSENGDFFLQDHEVVERVYQEYLQLLNVETEKEKQII*
Mol011408	MGRAPCCSKVGLHRGPWTAREDSLLISYIQNHGEGNWRSLPKKAGLLRCGKSCRLRWMNYLRPDIKRGNIGPQEEDLIIRLHCLLGNRWS<br>LIAGRLPGRTDNEIKNYWNSHLSKKLKKQGFAIREETPRPKRTATPNYHNKKKNNNNNSKLSETMQSDGAVENAKIYAPKPTRFKPTWSV

MEKSESVTEEYEKGSSDASSGTNYDVDLASWSHDDHGGEFDQTAVGFHDATHLDFAADFFMQDESLQRLYDEYSQLLQLEVGANQLAEP
LMQ*

Mol005657

MGIRPPCCDKLNVKKGLWTAEEDAKLLAYVSTHGSGNWTNVPKKAGLKRCGKSCRLRWTNYLRPNLKHEGFSSQEDELIVTLHATIGSR
WSIIANQLPGRTDNDVKNYWNTKLSKKLALNGIDPVTHRPISEIKHSITTLHFAAATAAGQPLPSPAATVGVGHHDSRLISINRDLKKILLSP
PIPPPLEPATPPVLWNASGQTAAAPRSFPPPPPPPPPPQDLEWVQFLAEDAFLCIDEHDAYRAPPFATVDADFDFMEEEADVSSFIDEMLDRDR
EIISEFSGLVGCHYAL*

Mol020055

MMRRPETTNAASSKNGATVHKLRKGLWSPEEDDKLMTYMMTNGQGCWTDVARNAGLQRCGKSCRLRWINYLRPDLKRGAFSIEEEELI
IHLHSILGNRWSQIAARLPGRTDNEIKNFWNSTIKKRLKNSSQPSPPSHDQKSPSDANKELIMEDIMFMRMNSSSSSSSSSMQAFSMNTINYN
LFPFPDTVANDCFINDMSSSLAHDHGMYNDGNAHGIIAMDGGLKVEEGYDHFFVPPLDSASQEENGANLDYAYQNYSRNIGEINSNMMTL
KDHDDTKNIIKGSHFAGSHEKWEEGQESMRVGEWSLEDLIEDVSSFPLLDFQVE*

Mol001669

MAWKKDSSKLNLSKSTPTLPLLRPQNLSFLKWFPSPLTLLPLTQLTTSFPSRQTLMRMKFWGKHNMVVAEEEISRDGGGENTNDSSGQSK
LCARGHWRPAEDSKLKELVAIYGPQNWNLIAEKLEGRSVFYRKSCRLRWFNQLDPRINRSAFTEEEEEKLMAAHRLYGNKWAMIARLFP
GRTDNAVKNHWHVIMARKYREQSTAYRRRKLSQTMHLRKAIIHFRSSRKTFADHRDQERTDIFRQNDNEVQALQSSSYSHQASILLTMHQ
SAGHLPHNISVASSSSGETPAHSAEVEVNRMDASHGHIERSISSSSPTDFLGSYLRE*

Mol006740

MGRPPCCDKPGIKKGPWTPEEDLILISYIQEHGPGNWRSVPINTDMTAASNVIAGLLRCSKSCRLRWTNYLRPGIKRGSFTPYEEGIIIHLQA
LLGNKWAAIASYLPQRTDNDIKNYWNTHLKKKLIKLQETIGSHNPLAIEAATVNTEFAGVQLQQQHPQYSSCTYASSTDNISRLLEGWMRS
SEKNTMQSSSIDIKNSGDSLICHEEFYHEKLPEMMPVTAEAVVEVEQNRSLTFLEKWLFDDGTGHVDGFMELPSDQLI*

Mol000510

MPEEDEKLREMVTRHGPHNWNAIAEKRRGRSGKSCGLRWFNQLDARIKRNTFKEEDEERLLSYHRIHGNRWAVIARLLPGRTDNAVKNH
WHVIMARRSRETSEVLKLNQFIRSEEKQRREDLEIKFSEDIFCPNLRGGDETKSYGYNSIFQGQGHQFFSCYPNVHIFEDGIKEESSIEFYDFL
QVNSSDSNATGKRSKMDEEEQEEHDKEKLESKGGVPFIDFLVDSSHDWST*

Mol001948

MGRQPCCDKVGLKKGPWTTEEDKKLINFILNNGQCCWRAVPKLAGLLRCGKSCRLRWTNYLRPDLKRGLLSESEEKLVIDLHSQLGNRW
SKIAANLPGRTDNEIKNHWNTHIKKKLRKMGIDPLTHKPLPSEDQLPHQTQHCEIPYAVSDLSTAVPLHRPLDSLEAFCTDDVPLMEPHEIIL
PFTPSTTPCSSSSTTERTAWPSSSSSSSSSSSVKAEEIFPSMEWSESIYLWGMNDFMGWDFISHEGDGKLSSTDLFNQNQEPWKFELF*

Mol002260

MGRQPCCDKLKVKKGPWTAEEDKKLVAFILNNGHCCWRAVPKLAGLLRCGKSCRLRWTNYLRPDLKRGLLTDAEEKIVIDLHASLGNR
WSKIASKLPGRTDNEIKNHWNTHIKKKLLKMGIDPVTHKLLNDQTSSTAMASPNSTVTDDEKFSKKHEPLDAEKVIINTVSQEESTNPQQN
CSNAIDRDEQVIGCLWDDDMPFIDKLWSSPISNEAWERSSEWLLDYQEFGIGDLELGRVESIGFADEQIKLN*

Mol023319

MTIIQDNSHQHMFQDASMPQHASTDGVILVPTPITALASQAISLYNRSHLDGWRKGPWTPQEDKLLTEHVKLHGEGRWNCVSRLTGLKRS
GKSCRLRWVNYLRPDLKRGKITPHEETIILELHAKWGNRWSIARSLPGRTDNEIKNYWRTHFKKGKKTTNIERARAKFLKQQQHQQLELE
VPQFEEIAITQWEIAYMCNMPYVFQGEEFTRKSIVSSDDIDWVMMRHIMPLGRIWNLDDLGLDMGDQASFLLLELDGIGVKKTV*

Mol013852

MSTGKKKQSPSIIELVFLSLCMSIARAPCCDKANVKKGPWSPEEDAKLKSYIDEHGTGGNWIALPHKIGLKRCGKSCRLRWLNYLRPNIKH
GGFSEEEDQIICNLFVSIGSRWSIIAAQLPGRTDNDIKNYWNTRLKKKLLGRRRESSSTSQHRHLSDDKDCNTKPNPNGSTQILTASALERLQ
LHMQIQGLHCPFSFYNNTSLWPKLYPLKNDCKIFQPLSTDATATSVHPLKLSQQAMEPIEQANISNSMHPNIQGSLGASSSSSSNLDIELHDL
LYGKESKLFSEIDSFNDLNIEEGTGWLGRNGFEEKSSSSSWDSAFALHPDSVLQEYGLGYDL*

Mol001734

MGRAPCCDKAIVKKGPWSPEEDAKLKAYIEEHGTGNNWIALPQKIGLKRCGKSCRLRWLNYLRPNIKHGGFSEEEDRIICSLYVSIGSRWSI
IAAQLPGRTDNDIKNYWNTRLKKKLLGRRRDLPPSQFSQLAPADQKLNDEGSNPNPNESSQNLTTSAIERLKLHIQLQGLHSPFSYGNASL
WPNNKLLQTLNSTDHSIATAASPLKHFQQTNICNSMISNMQEHIVIPSSSCNLEAELHNLLDGYKENYQFTQVDCLKEIMNDIDQKGFDWW
ESNEFVEKLSSTSWDSALQPDTVFIQDDELGYDQ*

Mol006579

MRPPTVVKKERASTTAVASGGAAAAPQQLLKKGPWTAAEDAILKEYVRKHGEGNWNALQKKSGLQRCGKSCRLRWVNHFRPNLKKES
FSPEEETLIIRLHAQFGNKWAHMAKHLRGRTDNEIKNYWNTRVKQRKRAGLPLYPPEIQQHISSRCHGQVQNAPWTANSPPTLKPLQPTNS
SFTSAMPLLSENPYGSILQDPSRGKNISFQFPFLTSPVLNWPENFDLGPPPLLVKEELPSSQFSSNYGDEQMPHCNSGLLNDLLPESHGEEKLL
SSTYLAIDLLGKLGYWDCPLGIKVIEEMLETDGRALIDMILTEMAMATPKTFVPDLYNNCSSSGSSGEFSNCPSSVTTDEEIELDMPQLEWS
YVHGTGGF*

Mol005587

MGRKPCCDKEGVKRGLWTIEEDQRLVDFILNNGIQCWRFVPKLAGLMRCGKSCRLRWINYLRPDLKRGALSEAEEEQIIQLHSNLGNRWS
KIASHLPGRTDNEIKNIWNTRIKKKLKLQQSLDQITNNIEVERENHISKVEDKGLELEISTGSSKSSLFLELEQNWVQEKNIKQANVSISSSAS
ASMDGNLNTSYWEGESNLHGGSNQLLWADTTDYFSSWDAYNCVDDFLRYENYP*

Mol016137

MEARKRDEFSKKRSWSSEEDEILLKYIESHGLRNWNIIGKNSGLSRDGKSCRLRWLNHLKPGLKKKLPFSKEEKFIIFSKHAALGNKWSKIA
KQLPGRTDNEIKNFINTHMKKCLKTGTIPIYPREISMKYAQTGTYGLPIASSDIADSVFQQWTELVDLPSIQNTEEDGKDIFLSSYLQNSQSSF
ESLTQPQLAAPQQALITGFSTVLSSEQCSNLSISALLYSDYSMNTDLYSLLSKFI*

Mol002877

MHSGGGSGRKELPETAAAIRKGPWMAEEDEVLMDFVKKHGPRDWSSIRSKGLLPRTGKSCRLRWVNKLKPDLKTSGCKFSAEEERIVIDL
QARFGNKWARIATCLPGRTDNDVKNFWSTRQKRLARLLQTALPMSIKPFDDDDHQAMIPFLQPIVTSCALLPYTLPCPDLIPFTEQYSNGQ
CSHVPYIEDPDSAKTLHMLNLDATALLIEPAIANSEICLQSSTPPQPPFDHSLLELPLLPEGQDFIPEFGDMNFPGHFGCAENSHAMQLHMAP
TFFDVDVGANDIKIEQPGTPESFFGDFPADMFDSLDQPPPS*

	MEGQYYGWGTITDGWRKGPWTPQEDQLLIEHVKLHGEGRWNSVSWLTDEQILWSGLKRSGKSSRLRWVNYLRPDLKRGKITAKEEAIIL
Mol017655	ELHARWGNRWSMIARSLPGRTDNEIKNYWRTHFKKSTKNVEKARARYLRLQQDQKQQLLLSRSNIMKEETSSQTQMAMMPKEMEEMTF
	LCNNMPLMLHEGGSSENQQPAVKIERAKRKLTTTHGGAFGI*
	MLGGVVRGLGFDCGACHVLLPVVLRFASFVWHLFLPFGLISSSLLGLVYSRNAVSGGFYIFMSVKIMLGLTFRFLNMCSVEKDGDTKMPP
	EEQPDSSSNDEGSWSGSPTSGEGIALKKGPWTSTEDAMLIDFVKKHGEGNWNAVQRNTGLPRCGKSCRLRWANHLRPDLKKGPLTREEE
	QFIIQMHAKIGNKWARMAALSVSFCNRNSYLSQCVTQLPGRTDNEIKNFWNTRTKRRQRAGLPSYPSNDDCSGASNENLLIQNASKLCHG
Mol016758	DIESSGVLQGSIYEMPRFPCTRPMSYAPLSDIHRTYMLSQELRPYTRCFLNQGANHTKRNIESEACLPGYHGSLGSVVSTFTELPSEHSESMY
	QNLGLGYSYDPDPDNKNLISFGCSIPGSHGSSHENLSASRCSSGTVKLELPSLQCPETNYSNWTAPPYHVDVPSPPENAPFLPDRASPRRNGL
	LEALVHESKTMNCGKKQSSEKSSCSSAITLSDMMESTAVNLCTEEWEETSDQMSPFSQSVTERAPITSNSFDEFHPKDPLGSEATMAVEEAS
	ATSLPDKEPSHWPDFLRPDALLESNWILTDSHRVKETSDTSFELLGEDLCTAMVSLDSCPWNSMPSVCQIPEYL*
	MAANDVDRIKGPWSPEEDEMLQMLVEHWSLISKSIPGRSGKSCRLRWFNQLSPKVEHRPFTPDEDETIISAHRRFGNKWATIARLLSALITP
Mol028701	LSHWNSTLERKEAAAAAAAWTSEERMMGLWRIAGRSSGRMAPGSVSARVGKVIKADAPDSTTGKWNPCDPFTFLTLSLPGSSCGQNESS
	DNQKQTDPQLLEKKPPSVSMASPFSLEFLEALQEIIHQEVKNYMSGLEHRGILPPPLPPEEDSKLNSAKGIGISRIN*
	MGRAPCCDKANVKRGPWSPEEDAALRSYVERHGTGGNWISLPQKAGQLTYACILSLFLFFKALNDAAKAAAFANYLRPDIKHGGFTEEE
Mol001134	DNIIFCLYKTIGSRWSVIASHLHGRTDNDVKNYWNTKLMKKIMAAGETINSPISNNQNTNKFHQPSPSPLLHHHQFKFRDWIQLVSTERRSS
	LTLQSSQWTTSSITVDDGGEGVTKPLPSSRSLVSPPGQLLGFVTSGTYESPTSLWANTDTKPQGVYQSLYS*
	MGRIPCCEKDNVKRGQWTPEEDNKLSSYIAQHGTRNWRLIPKNAGALLLDRWSLIAAQLSGRTDNDVKNHWNTKLKKKLSGMGIDPVT
	HKPFSHLMAEIATTLAPPQVAHLAEAALGCFKDEMLHLLTKRRIDFSSTYAVGVYPTPVPPPHSGEPNLVSDINGEETIQKIKMGLSRAVMQ
Mol012110	EPNPVKGWGSTANGEPDNHNLMCEMYPMAIDQGYMYGDPSAYVNDGEGSTWSTCNGGGGGSAAAQQHETGSRGYLLKGEEDDAEESE
	GVKGGSKEDAGVFGSECVLWDLSEDLMNHIV*
	MGRAPCCDKASVKKGPWSPEEDERLKSYIEKHGTGGNWIALPHKIGNFYFYAGLFMILIWSIIAAHLPGRTDNDIKNHWNTKLKTRLLGK
	QRKNQQNRRSCNLKQVMKKNKADNDQSASATTEANKAWLNLPISTITFHSTDHASYDNQSQTSISKLQMKLADVRLPCCGNGPHQVQVA
Mol014101	PSLRIPQAIYETPLNTMAYSSHENTLNDGSPLKNVGPEYFEQGFNYSNNNSLMKLEGFDFFYGMSSFINESINVSESITWSEMNSFTTSVLPIS
	STSEETLDFTSWETVKQC*

Mol018336	MGRAPCCDNMGVRRGPWTPEEDQILISHIQRYGHGNWRAIPKQAAGKLPGRTDNEIKNLWHGHLKKRFDPNQNMKEAKQKNRGNSKAK<br>EFKQATLEENVSSACSTTNDRDISSSVISTVGGENNSKTNMNYQLLQFDDSLCCFEPQSITKNCSLKLIFITDDFNPMSFYNGEGSNFWMNM<br>LGKAANLQDWQNM*
Mol018337	MGLERPWTPEEDQILISHIQRYGHGNWRAIPKQAGLVRCGKSCRLRWENHLRPGIKRGNFTKEEMDAVISLHAVLSSRHVFFSLHYCQWS<br>AIAGKLPGRTDNEIKNLWHGHLKKRFDPNQNMKDAKQKNRGNSKAKEFKQATLEENVSSACSTTNDRDMSSSVISTVGGENNSKTNMN<br>YQLLQFDDSLCCFEPQSITKNCSLNEIFITDDFNPMSFYNGEGSNFWMNMLGKAANLQDWQNM*
Mol013317	MHSGSSGGGVRCSRKELQSTETAAAIRKGPWMAEEDAVLIEYVKRYGPRDWSSIRSKGLLPRTGKSCRLRWLNKLRPDLKSGCKFSAEEE<br>WVVIDLQARFGNKWARIATYLPGRTDNDVKNFWSTRQKRLARLLQTPLPVGSRRNQGKVPMPPEAPMFEPNCNSGDLPYNDPQCLDLIPF<br>TAECANSKCIHPPFVEEPDAMKMVLMSSLDLPLSLLKPAITDRELCLPSSSSSAPQSPFDHSLPDLPLLSEGQGLILEFGEMSSPGQFCCAESS<br>QVMQLPPSSLLDLQFGDHGVKIEHPATPESFFGDFPADMFEFMDQPPVS*
Mol012133	MTLSVQLKEKPAVKPPETSAAVTGTTTEAPQSLKKGHWTAAEDAILKRYVEKYGEGNWNAVQKNLGLQRCGKSCRLRWANHLRPNLKK<br>GAFSPEEEALIVQLHAQLGNKWARMALQVLGLLL*
Mol021537	MAARCRDVDRIKGPWSPEEDEMLQILVVKHGARNWSLISKSIPGRSGKSCRLRWCNQLSPTVEHRPFTPEEDRTIISAHQRFGNKWATIAR<br>LLNGRTDNAIKNHWNSTLKRKYSGAALEDEGRSLKRSNSAGLSFSPGSPSSSDVSDSSHHSQPLMTPPPMVAPVYRPVPRTGCIVPSQLPHA<br>IDTDSSGTGSITISDPITSLTLSLPGSASDQSEASDHHLRQNQSDLLLLALPTKPEKLPLPAAAAFPFSNEFLVVMQEMIRQEVRNYLSGMEQR<br>ETMIMSPSHESLCNTVIKRIGISKID*
Mol011587	MGRLRVREEEPSEAKRVICAALWRPLYVEKDQSLVMQKPMQGEAFPDAQVYYYQIEGGRADEMDCASVNGGKSIETSSSSSVMESSIGA<br>VRGQWTAEEDRMLIRLVKQHGIRKWSQIARIFTGRIGKQCRERWHNHLRPDIKKDSWTEDEEILLVQAHKEIGNRWAEIAKRIPGRSENSIK<br>NHWNATKRKQSSKKRLRKMAFHGDTSTPKHTVLQGYIKSITSSLHEITPITSPSASHSRTSNEFQMKLLKVPQPHDSKLASQMVEELLSVEK<br>QEMRAFEKEEQDFLGCSDIEYVSQFLNTEEESFPSPSSNVTSAGFEAQSGSGGSHLQWDFYLANLLNRPSLPAIGEFGSSNAEMVTCVDGQA<br>SPIRSVGEMDLMEIYNWHLSHLPR*
Mol003679	MDGGSNEPFSPFKQMAQKPSWGFGTVLSEEMETKEHGRLNSKLCVRGHWRPAEDAKLKELVARYGPQNWNLIAEKLDGRSGKSCRLRW<br>FNQLDPRINRKAFTEEEEERLLSAHKFYGNKWALISRLFPGRTDNAVKNQWHVMMARRQRELSDACKRRKSSSSCFITSVKMEEKININSN<br>SSNECTSSRNFHLSMFLNSYKFPGTSVLIGFDGKPEASAAETVVAHEREKMSLPFIDFLGVGAT*

Mol015625	MAADKPEESKLCPRGHWRPGEDDKLRQLVQEFGPQNWNSIAEKLQGRSGKSCRLRWFNQLDPRINRRPFTEVEEERLLAAHRFHGNKWA<br>LIARLFPGRTDNAVKNHWHVIMARRQREKTKFLIDKSSFQQNHSHSHILNQQTCCSISPNQFNHYKYNYGILQSSHQPLQMFSNSSNIYWRF<br>GMLDGHMGCCRTVSGGVNQEEKDNDEALRSKNVVYIDFLGVGKD*
Mol023861	MENLDAVKHGEMKACPRGHWRPREDEKLRQLVEEFGPQNWNTISEKLQGRSGKSCRLRWFNQLNPRINRRPFTEEEEESLLTAHKIHGNK<br>WALIARLFPGRTDNAVKNHWHVIMARRQREKAKFLCKNGPSSTWPAFSNFSLTSERRVNYGILESSRFLNVPLLSFCDAYPACSLSGTGGS<br>GMIRNMNEVLRLGCNSRREILSINCRGGIQFLEEGNDEASWRREDLTYIDFLGVGKDC*
Mol017683	MSSDHAAAPAPAPAPSTQAKKDRHMVSWTQEEDDLLRAQVALHGSDNWTIVAAQFKDKTGRQCRRRWLTYLNTECKKGGWSQEEDML<br>LCEAQKIFGNRWTEIAKVVTGRTDNAVKNRFSTLRKKRAKHHVTSDENNNSCLHPSNKKIMIEPSANKQQNRYGDSSYVNSHESLTTSTH<br>DITGTQSRPPLAVLTKNFNNLSCVQAHCHLDSNTKLTACDKMHHLPLKQLQIVRSRAISLEGMIQNYCFATRELLSSLAVKACSENRNQSS<br>EDAWKSYMIWTREGGSLSSFSEMDFLLDNFEGVFEDFQCGDTEIQLSTRVVDLENSQTSSEISTGCTHDDPRNNVDKHQLNHCSVGCNEGE<br>SHQHEGISAPVKFVCQGGTMSPSLELTQNRENLLVSSKSEFDSPLKTIPPFHSFTEEIPSPEFSSSVSFDL*
Mol011807	MKERQRWRAEEDAILRAYVKQYGPREWNLVSQRMNVGLERDAKSCLERWKNYLKPGIKKGSLTEEEQRLVIHLQSKHGNKWKKIAAEV<br>PGRTAKRLGKWWEVFKEKQQRELKESNKISSTPIEPGKYDRILESFAEKLVKQRPVTPLLMASPLLPPWLASSNNTSSPSVALSLSPSTLTAA<br>PPTPWVQIERGVPENTLGLVNAQQNVTNSQQNLLGELVECCREIEEGRKEKIEEIESKVRELREEQKLALERIEAECWEQLLVVRRDAESKE<br>QKLAELWSAKHAALSKLIRQMTGHH*
Mol006728	MGLSLASPPSLFCSFENNGRINESGVLLEQQDGFFCGGKGSFVVEKNIHNMELEKRENETRLSKLCSRGHWRPAEDAKLRKLVALYGPQN<br>WNIISEKLDGRSGKSCRLRWFNQLDPRINRNAFTKEEEQKLLSAHKFYGNKWALIARLLP*
Mol009335	MDREWRKGKGAAWTREEDAKLTELCEAHDKRNWTVISSEIPGRSVAACRQRWINHLKPAIEHPPFTDEENAIIVGANQEQRKKWERIARL<br>LPGRTANAIRYHWKMNLCHLRNAPGSSQQAENL*

TABLE S2. Ka/Ks analysis of *CsMYB* genes

Subgroups	Gene pairs		Ka	Ks	Ka/Ks	Divergence time (mya)	Purify selection
S1	*Mol017692*	*Mol005176*	0.27	1.77	0.15	97.35	Yes
S1	*Mol018189*	*Mol017692*	0.16	1.46	0.11	80.19	Yes
S2	*Mol018337*	*Mol018336*	0.02	0.02	0.82	1.30	Yes
S2	*Mol011069*	*Mol018337*	0.43	1.06	0.40	58.51	Yes
S2	*Mol020712*	*Mol015023*	0.29	1.18	0.24	64.61	Yes
S3	*Mol010269*	*Mol010337*	0.35	1.91	0.18	104.97	Yes
S4	*Mol003893*	*Mol008437*	0.26	NaN	NaN	/	/
S4	*Mol008698*	*Mol009531*	0.18	1.35	0.14	73.96	Yes
S4	*Mol008437*	*Mol000543*	0.26	2.03	0.13	111.32	Yes
S4	*Mol004081*	*Mol019958*	0.22	1.92	0.11	105.70	Yes
S5	*Mol023305*	*Mol012178*	0.49	NaN	NaN	/	/
S6	*Mol011449*	*Mol028264*	0.04	0.05	0.66	3.00	Yes
S10	*Mol010343*	*Mol001350*	0.24	0.97	0.25	53.36	Yes
S11	*Mol013646*	*Mol008801*	0.05	0.13	0.37	7.08	Yes
S11	*Mol008801*	*Mol008800*	0.16	0.53	0.30	29.17	Yes
S13	*Mol002856*	*Mol018126*	0.34	3.26	0.10	178.98	Yes
S14	*Mol013852*	*Mol001734*	0.19	1.01	0.18	55.54	Yes
S14	*Mol019578*	*Mol000176*	0.24	1.48	0.16	81.28	Yes
S17	*Mol023320*	*Mol023319*	0.11	0.09	1.15	5.17	No
S17	*Mol021224*	*Mol019840*	0.23	0.96	0.24	52.84	Yes
S17	*Mol023319*	*Mol017655*	0.24	1.15	0.21	63.16	Yes
S18	*Mol012133*	*Mol006579*	0.23	NaN	NaN	/	/
S18	*Mol017152*	*Mol016758*	0.35	0.86	0.41	47.26	Yes
S18	*Mol012137*	*Mol012133*	0.12	0.37	0.31	20.40	Yes

S19	*Mol010957*	*Mol012057*	0.16	1.06	0.15	58.45	Yes
S20	*Mol002558*	*Mol007990*	0.22	1.22	0.18	67.16	Yes
S21	*Mol003679*	*Mol022732*	0.32	0.98	0.32	53.86	Yes
S21	*Mol011788*	*Mol000510*	0.28	1.09	0.26	59.85	Yes
S21	*Mol028471*	*Mol001669*	0.28	1.16	0.24	63.88	Yes
S21	*Mol000510*	*Mol018713*	0.36	2.11	0.17	115.89	Yes
S21	*Mol023861*	*Mol015625*	0.22	1.70	0.13	93.44	Yes
S22	*Mol028701*	*Mol013522*	0.06	0.12	0.55	6.42	Yes
S22	*Mol013522*	*Mol021537*	0.26	1.31	0.20	72.23	Yes
	Mol002877	*Mol013317*	0.20	0.70	0.29	38.70	Yes
	Mol016709	*Mol000639*	0.32	1.22	0.26	66.77	Yes
	Mol010990	*Mol016869*	0.45	NaN	NaN	/	/

TABLE S3. Protein secondary structure prediction of *DrMYBs* and *CsMYBs*.

Subgroups	Gene ID	Alpha helix (%)	Extended strand (%)	Beta turn (%)	Random coil (%)
S1	*AtMYB96*	29.26	5.4	2.56	62.78
S1	*Mol005176*	29.39	6.07	2.88	61.66
S1	*Mol017692*	32.33	4.67	3.33	59.67
S1	*Mol018189*	35.03	6.05	4.14	54.78
S1	*OsMYB60*	36.98	6.43	2.89	53.7
S1	*AtMYB60*	39.64	5.71	2.5	52.14
S1	*Mol006363*	37.54	6.83	4.1	51.54
S1	*Mol006740*	42.75	4.58	4.2	48.47
S2	*Mol018337*	30.71	4.56	4.15	60.58
S2	*Mol020712*	32.34	4.26	5.11	58.3
S2	*Mol013321*	35.62	3.86	5.58	54.94
S2	*OsMYB4*	35.41	4.67	5.06	54.86
S2	*Mol011069*	35.78	5.6	4.74	53.88
S2	*Mol015023*	37.96	3.67	4.9	53.47
S2	*Mol018336*	41.45	3.63	3.63	51.3
S3	*Mol010269*	38.87	9.43	4.53	47.17
S3	*Mol001517*	35.79	13.11	5.74	45.36
S3	*Mol010337*	43.45	8.61	5.62	42.32
S4	*Mol003893*	30.25	7.56	5.88	56.3
S4	*Mol008437*	31.34	5.07	7.37	56.22
S4	*Mol008698*	32.06	7.25	7.25	53.44
S4	*Mol019958*	29.72	8.96	8.49	52.83
S4	*Mol009531*	36.7	4.59	7.34	51.38
S4	*Mol004081*	34.66	11.16	5.18	49

S4	*Mol000543*	32.27	12.75	6.77	48.21
S5	*Mol023305*	36.71	2.53	8.02	52.74
S5	*Mol012178*	42.58	12.11	7.81	37.5
S6	*Mol011449*	43.12	2.29	7.34	47.25
S6	*Mol028264*	45.26	9.91	4.31	40.52
S7	*Mol008232*	40.72	14.66	5.54	39.09
S7	*Mol008979*	41.05	13.89	11.11	33.95
S8	*Mol001948*	29.74	6.32	4.09	59.85
S8	*Mol002260*	36.25	4.78	3.98	54.98
S8	*Mol005587*	43.04	7.17	5.91	43.88
S9	*Mol010990*	30.81	9.52	3.08	56.58
S9	*Mol016869*	36.3	7.78	5.56	50.37
S10	*Mol001512*	43.08	4.31	4.62	48
S10	*Mol010343*	39.58	12.39	3.93	44.11
S10	*Mol001350*	42.55	11.38	5.15	40.92
S11	*Mol008800*	33.76	6.11	4.5	55.63
S11	*Mol008801*	41.88	4.87	5.84	47.4
S11	*Mol013646*	35.11	7.63	11.45	45.8
S13	*Mol018126*	24.88	4.88	4.39	65.85
S13	*Mol002856*	29.5	6.27	5.74	58.49
S13	*Mol014116*	35.57	6.72	8.7	49.01
S14	*Mol014101*	33.68	8.59	3.09	54.64
S14	*Mol013852*	35.99	6.78	3.24	53.98
S14	*Mol007569*	34.48	7.76	3.88	53.88
S14	*Mol001134*	31.75	11.51	5.56	51.19
S14	*Mol001734*	39.61	6.17	4.22	50

S14	*Mol000176*	38.83	9.39	3.24	48.54
S14	*Mol019578*	37.19	9.12	5.26	48.42
S14	*Mol004798*	28.62	17.39	8.7	45.29
S17	*Mol019840*	36.96	7.83	6.52	48.7
S17	*Mol026352*	36.94	8.28	7.64	47.13
S17	*Mol023318*	32.52	14.63	12.2	40.65
S17	*Mol021224*	34.43	16.39	10.16	39.02
S17	*Mol023319*	44.32	10.98	6.82	37.88
S17	*Mol023320*	46.05	8.37	7.91	37.67
S17	*Mol006174*	50.89	7.14	5.36	36.61
S17	*Mol017655*	44.95	13.76	8.72	32.57
S18	*Mol017152*	22.74	6.32	3.79	67.15
S18	*AtMYB33*	25.38	4.81	3.65	66.15
S18	*Mol014836*	28.31	5.03	2.05	64.62
S18	*AtMYB101*	28.16	4.9	4.69	62.24
S18	*Mol016758*	26.25	9.34	3.54	60.87
S18	*Mol012137*	38.4	3.99	4.49	53.12
S18	*Mol006579*	33.6	7.8	5.65	52.96
S18	*Mol012133*	45.08	9.84	8.2	36.89
S19	*Mol012057*	38	4.5	4.5	53
S19	*Mol010957*	31.53	12.32	4.93	51.23
S20	*AtMYB2*	32.97	9.52	2.93	54.58
S20	*Mol007990*	34.66	6.86	3.97	54.51
S20	*TaPIMP1*	46.13	3.1	4.02	46.75
S20	*Mol002558*	43.48	5.53	5.93	45.06
S20	*Mol015419*	39.05	10.48	10	40.48

S20	*OsMYB2*	49.67	9.67	4.67	36
S21	*Mol014848*	22.72	11.24	7.49	58.55
S21	*Mol009191*	31.31	8.63	5.43	54.63
S21	*Mol023861*	35.15	5.86	4.6	54.39
S21	*Mol011788*	31.78	9.75	4.66	53.81
S21	*Mol000510*	32.19	11.16	4.29	52.36
S21	*Mol003679*	35.8	8.64	6.17	49.38
S21	*Mol015625*	41.78	4.89	4.44	48.89
S21	*Mol022732*	37.3	13.18	4.5	45.02
S21	*Mol018713*	38.59	12.86	4.15	44.4
S21	*Mol001669*	39.88	11.35	5.52	43.25
S21	*Mol028471*	42.74	10.26	8.97	38.03
S21	*Mol006728*	38.67	16.67	11.33	33.33
S22	*Mol021537*	26.07	7.26	3.63	63.04
S22	*AtMYB44*	21.97	10.82	4.92	62.3
S22	*TaMYB70*	23.95	9.28	5.69	61.08
S22	*Mol013522*	32.76	6.21	3.79	57.24
S22	*Mol028701*	35.66	5.81	3.1	55.43
S22	*Mol017415*	30.99	10.74	6.2	52.07
S22	*Mol009335*	49.59	8.13	7.32	34.96
S23	*Mol016109*	23.94	6.91	3.72	65.43
S24	*Mol003923*	33.45	3.45	4.48	58.62
S25	*Mol011587*	39.85	6.94	3.34	49.87
S25	*Mol001031*	41.91	9.25	2.89	45.95
	Mol002877	28.25	4.44	3.81	63.49
	Mol013317	27.24	6.81	4.95	60.99

Mol000639	24.07	11.11	6.67	58.15
Mol011408	29.78	6.25	5.88	58.09
Mol020055	29.23	8.62	5.23	56.92
Mol017976	34.7	4.1	5.05	56.15
Mol004182	31.01	8.54	5.06	55.38
Mol017683	34.65	8.84	5.12	51.4
Mol012110	36.24	6.38	6.38	51.01
Mol006542	31.07	11	7.12	50.81
Mol016709	37.98	6.59	9.69	45.74
Mol005657	43.64	4.81	5.84	45.7
Mol016137	39	9.96	6.22	44.81
Mol011807	57.05	8.72	3.36	30.87

TABLE S4a. Putative *cis*-acting elements identified in the promoter regions of *CsMYB* genes

	Cis-acting elements	Number	Function
Light responsive	3-AF1 binding site	7	light responsive element
	ACE	10	cis-acting element involved in light responsiveness
	AE-box	26	part of a module for light response
	AT1-motif	12	part of a light responsive module
	ATCT-motif	30	part of a conserved DNA module involved in light responsiveness
	Box 4	441	part of a conserved DNA module involved in light responsiveness
	chs-CMA1a	22	part of a light responsive element
	chs-CMA2a	9	part of a light responsive element
	GA-motif	30	part of a light responsive element
	Gap-box	10	part of a light responsive element
	GATA-motif	72	part of a light responsive element
	G-box	176	cis-acting regulatory element involved in light responsiveness
	GT1-motif	102	light responsive element
	I-box	35	part of a light responsive element
	LAMP-element	16	part of a light responsive element
	MRE	40	MYB binding site involved in light responsiveness
	Sp1	23	light responsive element
	TCCC-motif	41	part of a light responsive element
	TCT-motif	91	part of a light responsive element
Hormone	ABRE	151	cis-acting element involved in the abscisic acid responsiveness
	AuxRR-core	13	cis-acting regulatory element involved in auxin responsiveness
	CGTCA-motif	151	cis-acting regulatory element involved in the MeJA-responsiveness
	GARE-motif	14	gibberellin-responsive element

	P-box	19	gibberellin-responsive element
	TATC-box	20	cis-acting element involved in gibberellin-responsiveness
	TCA-element	58	cis-acting element involved in salicylic acid responsiveness
	TGACG-motif	151	cis-acting regulatory element involved in the MeJA-responsiveness
	TGA-element	31	auxin-responsive element
Abiotic/biotic stress	ARE	163	cis-acting regulatory element essential for the anaerobic induction
	GC-motif	7	enhancer-like element involved in anoxic specific inducibility
	LTR	39	cis-acting element involved in low-temperature responsiveness
	MBS	48	MYB binding site involved in drought-inducibility
	TC-rich repeats	40	cis-acting element involved in defense and stress responsiveness
Development	CAT-box	52	cis-acting regulatory element related to meristem expression
	circadian	31	cis-acting regulatory element involved in circadian control
	GCN4_motif	23	cis-regulatory element involved in endosperm expression
	O2-site	49	cis-acting regulatory element involved in zein metabolism regulation
	RY-element	12	cis-acting regulatory element involved in seed-specific regulation

TABLE S4b. The number (No.) of MBS elements in *CsMYBs* and 11 *DsMYBs*.

Subgroups	Gene ID	No. of MBS elements	Subgroups	Gene ID	No. of MBS elements
S1	*OsMYB60*	1	S14	*Mol019578*	1
S1	*AtMYB96*	1	S14	*Mol000176*	
S1	*Mol006740*		S14	*Mol004798*	
S1	*AtMYB60*		S14	*Mol014101*	
S1	*Mol006363*		S14	*Mol013852*	
S1	*Mol018189*		S14	*Mol001734*	
S1	*Mol017692*		S14	*Mol007569*	
S1	*Mol005176*		S14	*Mol001134*	
S2	*Mol015023*	1	S17	*Mol006174*	1
S2	*Mol011069*		S17	*Mol021224*	1
S2	*Mol013321*		S17	*Mol023318*	
S2	*Mol018337*		S17	*Mol023320*	
S2	*Mol018336*		S17	*Mol023319*	
S2	*Mol020712*		S17	*Mol017655*	
S2	*OsMYB4*		S17	*Mol026352*	
S3	*Mol001517*		S17	*Mol019840*	
S3	*Mol010269*		S18	*AtMYB101*	1
S3	*Mol010337*		S18	*Mol017152*	1
S4	*Mol003893*	2	S18	*Mol016758*	1
S4	*Mol008437*	2	S18	*Mol014836*	1
S4	*Mol000543*	2	S18	*Mol012137*	
S4	*Mol004081*	1	S18	*Mol012133*	
S4	*Mol008698*		S18	*Mol006579*	
S4	*Mol009531*		S18	*AtMYB33*	
S4	*Mol019958*		S19	*Mol010957*	
S5	*Mol012178*	1	S19	*Mol012057*	
S5	*Mol023305*		S20	*Mol015419*	3
S6	*Mol011449*	2	S20	*AtMYB2*	1
S6	*Mol028264*		S20	*TaPIMP1*	1
S7	*Mol008232*	2	S20	*OsMYB2*	1
S7	*Mol008979*		S20	*Mol002558*	
S8	*Mol001948*	1	S20	*Mol007990*	
S8	*Mol002260*	1	S21	*Mol006728*	1
S8	*Mol005587*		S21	*Mol028471*	1
S9	*Mol010990*		S21	*Mol001669*	1
S9	*Mol016869*		S21	*Mol011788*	1
S10	*Mol001512*		S21	*Mol000510*	1
S10	*Mol010343*		S21	*Mol018713*	1
S10	*Mol001350*		S21	*Mol003679*	
S11	*Mol008800*	2	S21	*Mol014848*	

S11	*Mol008801*	1	S21	*Mol022732*	
S11	*Mol013646*		S21	*Mol009191*	
S13	*Mol002856*		S21	*Mol015625*	
S13	*Mol018126*		S21	*Mol023861*	
S13	*Mol014116*		S22	*TaMYB70*	2
S10	*Mol001512*		S22	*Mol017415*	2
S10	*Mol010343*		S22	*Mol009335*	2
S10	*Mol001350*		S22	*Mol021537*	1
S11	*Mol008800*	2	S22	*AtMYB44*	1
S11	*Mol008801*	1	S22	*Mol028701*	
S11	*Mol013646*		S22	*Mol013522*	
S13	*Mol002856*		S23	*Mol016109*	1
S13	*Mol018126*		S24	*Mol003923*	
S13	*Mol014116*		S25	*Mol001031*	1
S10	*Mol001512*		S25	*Mol011587*	
S10	*Mol010343*			*Mol006542*	3
S10	*Mol001350*			*Mol002877*	2
S11	*Mol008800*	2		*Mol000639*	1
S11	*Mol008801*	1		*Mol011807*	1
S11	*Mol013646*			*Mol016137*	1
S13	*Mol002856*			*Mol017976*	
S13	*Mol018126*			*Mol004182*	
S13	*Mol014116*			*Mol020055*	
	Mol005657			*Mol016709*	
	Mol012110			*Mol017683*	
	Mol011408			*Mol013317*	

TABLE S5a. Expression profiles of *CsMYB* genes in leaves

Genes	L1	L2	L3
Mol009531	150.12	149.09	86.66
Mol008698	86.98	199.03	95.21
Mol001948	69.57	153.43	11.58
Mol002260	67.15	287.74	7.18
Mol019958	54.06	56.99	16
Mol018189	43.79	14.21	38.25
Mol020055	43.34	95.69	0.97
Mol005176	42.44	64.78	36.82
Mol021537	32.64	91.16	72.31
Mol018713	29.75	39.38	0.25
Mol006579	28.57	26.32	22.59
Mol001517	24.85	87.73	0.39
Mol021224	21.33	21.15	37.37
Mol017415	16.97	8.86	5.5
Mol017655	16.48	58.32	70.31
Mol010269	15.67	100.92	4.6
Mol004182	13.31	14.47	0.09
Mol011788	11.97	20.69	5.33
Mol016109	10.75	13.54	11.94
Mol003893	10.55	12.86	9.82
Mol004081	10.26	10.27	2.03
Mol007990	9.34	60.4	57.12
Mol008437	8.31	14.41	4.56
Mol002856	7.92	2.56	3.17
Mol007569	7.39	7.23	3.55
Mol016869	6.94	2.08	10.65
Mol026352	5.51	10.45	3.63
Mol000176	4.39	31.66	5.16
Mol016709	2.72	7.86	1.74
Mol010337	1.81	10.42	0.44
Mol015419	1.52	89.42	57.79
Mol011408	1.11	10.41	5.66
Mol004798	0.22	9.59	2.64
Mol011449	0.15	5.15	0
Mol015023	0	5.9	3.7

TABLE S5b. Expression profiles of *CsMYB* genes in roots

Genes	R1	R2	R3
Mol001948	146.64	1.74	3.12
Mol008698	144.73	63.48	28.87
Mol015419	118.33	42.14	7.82
Mol007990	94.86	26.08	8.38
Mol003923	91.85	44.66	24.89
Mol001517	86.75	9.61	7.51
Mol009531	80.04	38.69	16.39
Mol019840	68.84	38.74	11.72
Mol006174	66.79	120.33	4.01
Mol002856	58.53	29.27	9.72
Mol021537	51.7	134.36	787.3
Mol010269	51.23	41.04	9.06
Mol013852	45.72	8.95	5.4
Mol023318	38.09	64.81	3.39
Mol011788	36.9	5.4	5.29
Mol002260	35.33	22.27	11.93
Mol004081	35.33	18.87	9.29
Mol026352	35.31	22.63	1.1
Mol020055	35.29	0	0
Mol017655	32.04	3.13	7.29
Mol006579	31.25	83.86	125.33
Mol019958	30.19	54.59	25.29
Mol003893	30.02	101.56	112.83
Mol021224	27.47	48.53	44.98
Mol005176	26.68	28.25	25.01
Mol011408	25.71	6.29	7.42
Mol008437	25.2	5.71	5.09
Mol004798	21.12	5.2	3.13
Mol001734	18.92	4.35	1.98
Mol017976	15.68	4.59	7.64
Mol016869	14.91	16.15	18.54
Mol019578	14.43	62.68	35.12
Mol000176	13.74	3.15	1.57
Mol016109	13.28	25.57	19.75
Mol014101	13.2	18.11	9.17
Mol018713	10.21	1.06	1.85
Mol016709	9.91	1.22	3.04
Mol007569	9.85	39.58	32.44
Mol001512	9.66	1.95	1.95
Mol010337	9.16	0.94	1.63
Mol000639	6.57	0.23	0.69
Mol015023	5.8	0.39	0.26

Mol010343	4.29	5.19	3.45
Mol018189	3.54	5.61	12.65
Mol008801	2.92	8.41	9.67
Mol013522	2.81	7.42	15.57
Mol023320	1.86	7.39	0.75
Mol011807	1.25	3.43	5.93
Mol010990	0.85	8.94	4.17

TABLE S6a. The fold-changes of *CsMYB* genes in leaves of *C. sinense* under drought stress treatment

Subfamilies	Gene ID	Fold change of L2	Regulation	Fold change of L3	Regulation
S21	*Mol011788*	1.728487886		0.445279866	down
S21	*Mol018713*	1.323697479		0.008403361	down
S22	*Mol021537*	2.792892157	up	2.215379902	up
S22	*Mol017415*	0.52209782		0.324101355	down
S23	*Mol016109*	1.259534884		1.110697674	
S18	*Mol006579*	0.921246062		0.790689534	
S17	*Mol017655*	3.538834951	up	4.266383495	up
S17	*Mol021224*	0.991561181		1.751992499	
S17	*Mol026352*	1.896551724		0.658802178	
S20	*Mol015419*	58.82894737	up	38.01973684	up
S20	*Mol007990*	6.466809422	up	6.115631692	up
S13	*Mol002856*	0.323232323	down	0.400252525	down
	Mol004182	1.087152517		0.006761833	down
S16	*Mol011449*	34.33333333	up	0	
	Mol020055	2.207891094	up	0.022381172	down
S8	*Mol001948*	2.205404628	up	0.166451056	down
S8	*Mol002260*	4.285033507	up	0.106924795	down
S4	*Mol008698*	2.288227179	up	1.094619453	
S4	*Mol004081*	1.000974659		0.19785575	down
S4	*Mol009531*	0.993138822		0.577271516	
S4	*Mol019958*	1.054199038		0.295967444	down
S4	*Mol003893*	1.218957346		0.930805687	
S4	*Mol008437*	1.734055355		0.548736462	
S9	*Mol016869*	0.299711816	down	1.534582133	

	Mol011408	9.378378378	up	5.099099099	up
	Mol016709	2.889705882	up	0.639705882	
S14	*Mol000176*	7.211845103	up	1.175398633	
S14	*Mol004798*	43.59090909	up	12	up
S14	*Mol007569*	0.97834912		0.48037889	down
S1	*Mol018189*	0.324503311	down	0.873487098	
S1	*Mol005176*	1.526390198		0.867577757	
S3	*Mol010337*	5.756906077	up	0.243093923	down
S3	*Mol001517*	3.530382294	up	0.015694165	down
S3	*Mol010269*	6.440331844	up	0.293554563	down
S2	*Mol015023*	NaN		NaN	

TABLE S6b. The fold-changes of *CsMYB* genes in roots of *C. sinense* under drought stress treatment

Subfamilies	Gene ID	R2/R1	Regulation	R3/R1	Regulation
S21	*Mol011788*	0.146341463	down	0.143360434	down
S21	*Mol018713*	0.103819785	down	0.181194907	down
S22	*Mol013522*	2.640569395	up	5.540925267	up
S22	*Mol021537*	2.598839458	up	15.22823985	up
S23	*Mol016109*	1.925451807		1.487198795	
	Mol011807	2.744	up	4.744	up
S18	*Mol006579*	2.68352	up	4.01056	up
S17	*Mol023320*	3.97311828	up	0.403225806	down
S17	*Mol006174*	1.801617009		0.060038928	down
S17	*Mol023318*	1.701496456		0.088999737	down
S17	*Mol017655*	0.097690387	down	0.22752809	down
S17	*Mol021224*	1.766654532		1.637422643	
S17	*Mol026352*	0.640894931		0.031152648	down
S17	*Mol019840*	0.562754213		0.170249855	down
S20	*Mol015419*	0.356122708	down	0.066086369	down
S20	*Mol007990*	0.274931478	down	0.088340713	down
S16	*Mol002856*	0.500085426		0.166068683	down
	Mol017976	0.292729592	down	0.487244898	down
	Mol020055	0		0	
S8	*Mol001948*	0.011865794	down	0.021276596	down
S8	*Mol002260*	0.630342485		0.337673365	down
S4	*Mol008698*	0.438609825	down	0.199474884	down
S4	*Mol004081*	0.534106991		0.262949335	down
S4	*Mol009531*	0.483383308	down	0.204772614	down

S4	*Mol019958*	1.808214641		0.837694601	
S4	*Mol003893*	3.383077948	up	3.758494337	up
S4	*Mol008437*	0.226587302	down	0.201984127	down
S11	*Mol008801*	2.880136986	up	3.311643836	up
S9	*Mol016869*	1.083165661		1.243460765	
S9	*Mol010990*	10.51764706	up	4.905882353	up
S10	*Mol010343*	1.20979021		0.804195804	
S10	*Mol001512*	0.201863354	down	0.201863354	down
S24	*Mol003923*	0.486227545	down	0.270985302	down
S14	*Mol011408*	0.244651886	down	0.288603656	down
S14	*Mol016709*	0.123107972	down	0.306760848	down
S14	*Mol000639*	0.03500761	down	0.105022831	down
S14	*Mol019578*	4.343728344	up	2.433818434	up
S14	*Mol000176*	0.229257642	down	0.11426492	down
S14	*Mol013852*	0.19575678	down	0.118110236	down
S14	*Mol001734*	0.229915433	down	0.104651163	down
S14	*Mol004798*	0.246212121	down	0.148200758	down
S14	*Mol014101*	1.371969697		0.69469697	
S14	*Mol007569*	4.018274112	up	3.293401015	up
S1	*Mol018189*	1.584745763		3.573446328	up
S1	*Mol005176*	1.058845577		0.937406297	
S3	*Mol010337*	0.102620087	down	0.177947598	down
S3	*Mol001517*	0.110778098	down	0.086570605	down
S3	*Mol010269*	0.80109311		0.176849502	down
S2	*Mol015023*	0.067241379	down	0.044827586	down

TABLE S7. The primers of *CsMYB* genes.

Gene ID	Primer name	Sequence
Mol021537	37-F	TCTAGCTCTACCAACCAAACCG
	37-R	TCCCGCTCAGATAATTCCTCA
Mol018189	89-F	ACAGTCGTTCACCATCGTCG
	89-R	TAGGCGGTTTCCTCATCCA
Mol015419	19-F	GGAAACCGCTGGTCAAAGA
	19-R	GGACGATGAGAACCCATTACG
Mol002260	60-F	TTGTTGTTGGCGGGCAGTA
	60-R	TCGGGGCGAAGATAGTTTGT
Mol018713	13-F	ATTTCCTGGTCGCACAGATAAC
	13-R	AAACTGCCTTGCTTCCCTTG
Mol001948	48-F	CAGGCTGAGATGGACGAACTAT
	48-R	CGGTTCTTCCAGGGAGGTTT
Mol008698	98-F	CTCGGCAACAAATGGTCGT
	98-R	GCTGCTCTTGTTTCTTTCGTCT
Mol000176	76-F	GGCTCCGATGGCTGAACTAT
	76-R	TGATCTTTGCGGGATTTGC
Mol010269	69-F	AATAAGGGCTCATGGACGGT
	69-R	CGACAACTCTTCCCACAACG
Mol022529	ACT-F	TGGCATCACACCTTCTACAAC
	ACT-R	CATAGCAGGCACATTGAAAGTC

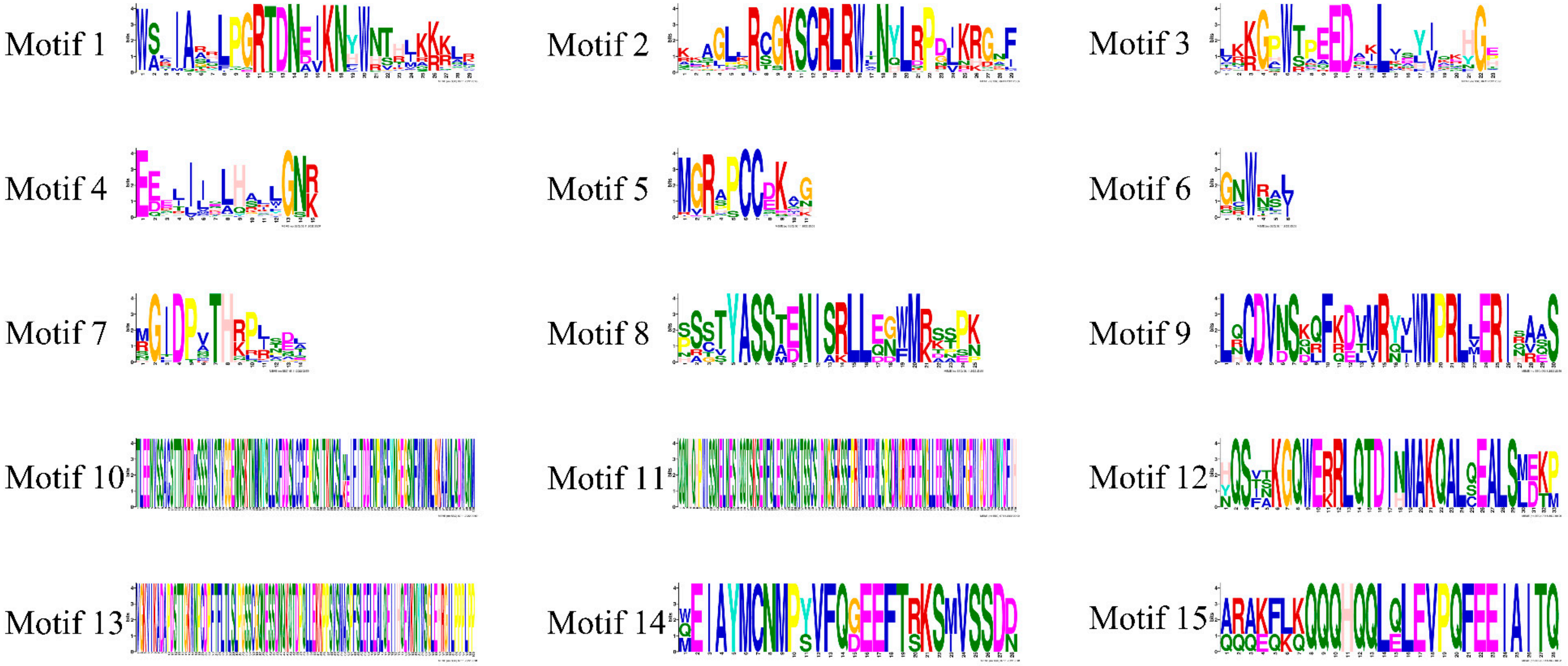

FIGURE S1. The 15 motifs of *CsMYBs* and *DrMYBs*

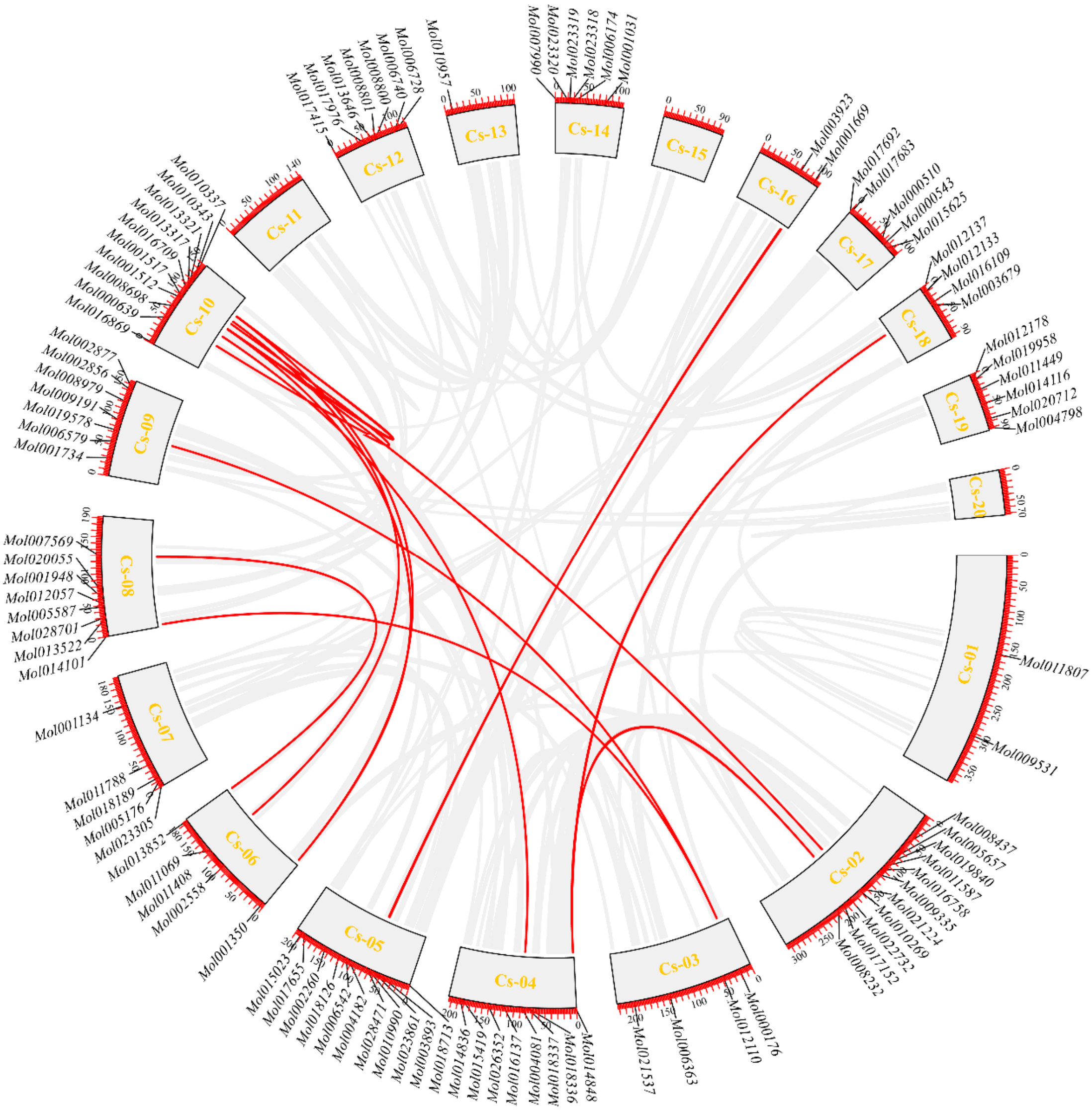

FIGURE S2. The collinearity of *CsMYB* genes in *C. sinensis*. The gray line in the background indicates collinear blocks within species, while the red lines highlight the syntenic R2R3-MYB gene pairs.

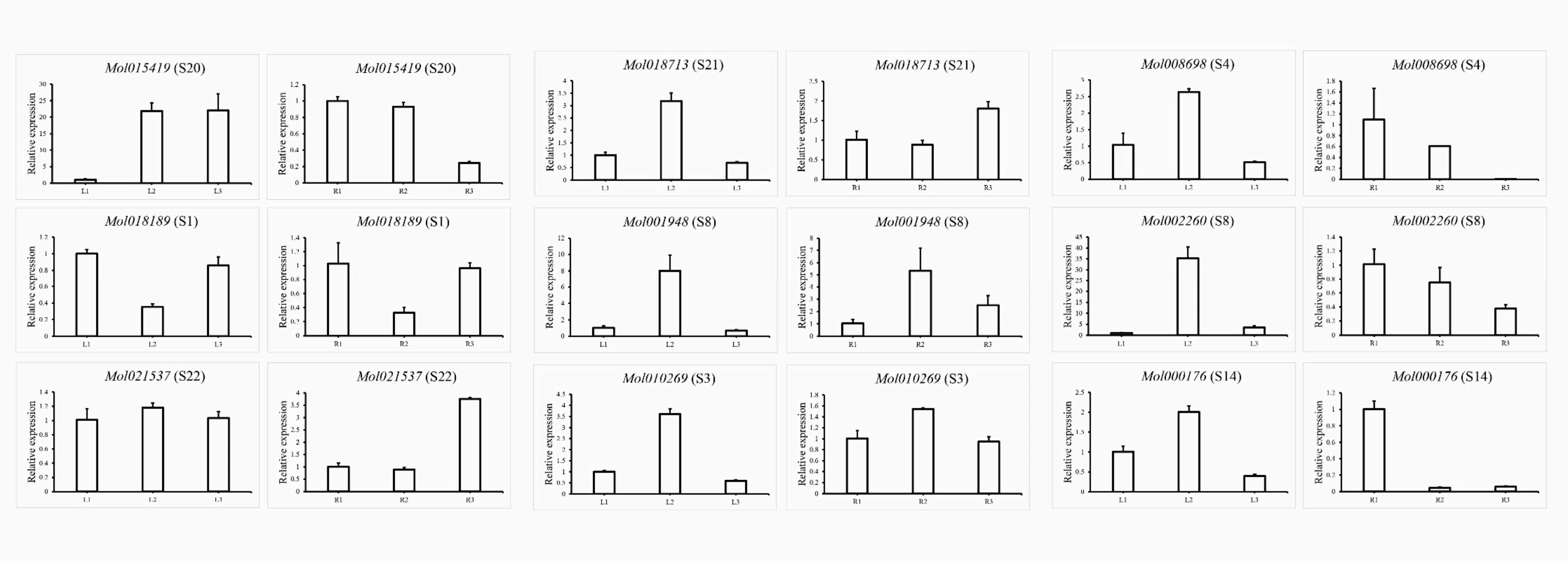

Figure S3. RT–qPCR validation of transcriptomic data of nine *CsMYB* genes under drought stress.

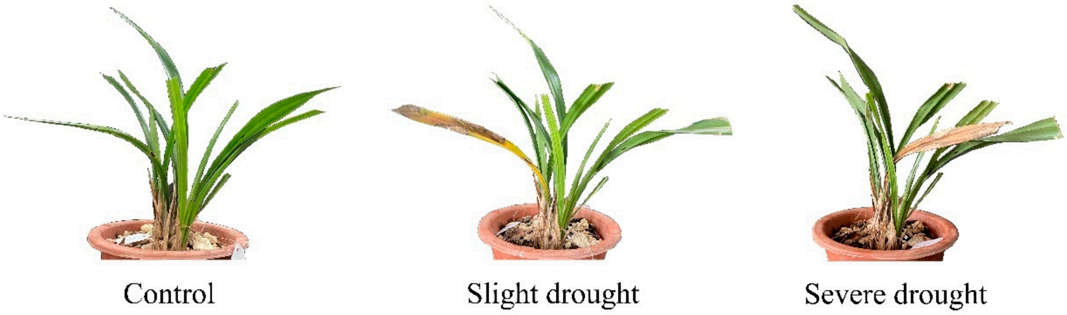

FIGURE S4. The plant of *C. sinense* under three treatments.
